# Phase separation propensity of the intrinsically disordered AB region of human RXRβ

**DOI:** 10.1186/s12964-023-01113-4

**Published:** 2023-05-04

**Authors:** Katarzyna Sołtys, Andrzej Ożyhar

**Affiliations:** Department of Biochemistry, Molecular Biology and Biotechnology, Faculty of Chemistry, Wrocław University of Science and Technology, Wybrzeże Wyspiańskiego 27, 50-370 Wrocław, Poland

**Keywords:** Nuclear receptor, Retinoid x receptor subtype beta, AB region, Intrinsically disordered region, Liquid–liquid phase separation, Liquid condensates

## Abstract

RXRβ is one of three subtypes of human retinoid X receptor (RXR), a transcription factor that belongs to the nuclear receptor superfamily. Its expression can be detected in almost all tissues. In contrast to other subtypes – RXRα and RXRγ – RXRβ has the longest and unique N-terminal sequence called the AB region, which harbors a ligand-independent activation function. In contrast to the functional properties of this sequence, the molecular properties of the AB region of human RXRβ (AB_*h*RXRB) have not yet been characterized. Here, we present a systematic biochemical and biophysical analysis of recombinant AB_*h*RXRB, along with in silico examinations, which demonstrate that AB_*h*RXRB exhibits properties of a coil-like intrinsically disordered region. AB_*h*RXRB possesses a flexible structure that is able to adopt a more ordered conformation under the influence of different environmental factors. Interestingly, AB_*h*RXRB promotes the formation of liquid–liquid phase separation (LLPS), a phenomenon previously observed for the AB region of another human subtype of RXR – RXRγ (AB_*h*RXRG). Although both AB regions seem to be similar in terms of their ability to induce phase separation, they clearly differ in the sensitivity to factors driving and regulating LLPS. This distinct LLPS response to environmental factors driven by the unique amino acid compositions of AB_*h*RXRB and AB_*h*RXRG can be significant for the specific modulation of the transcriptional activation of target genes by different subtypes of RXR.

Video Abstract

**Supplementary Information:**

The online version contains supplementary material available at 10.1186/s12964-023-01113-4.

## Introduction

Retinoid X receptors (RXRs) are members of the nuclear receptor (NR) superfamily of ligand-dependent transcription factors. They were first described in 1992 by Mangelsdorf et al. as NRs able to respond to derivatives of vitamin A [1]. RXRs serve multiple functions in the gene regulation of mammalian signaling systems. Through the ability to form homodimers, homotetramers and heterodimers with a diverse range of NRs (e.g., peroxisome proliferator-activated receptor (PPAR), liver X receptor (LXR), vitamin D receptor (VDR) and retinoic acid receptor (RAR)), they regulate and coordinate multiple processes, including cell differentiation, development, the immune response, and lipid and glucose metabolism [2]. RXR-controlled transcriptional programs might be potential targets for the treatment of pathologies such as insulin resistance, autoimmunity, atherosclerosis and neurodegeneration [3, 4].

**Fig. 1:**
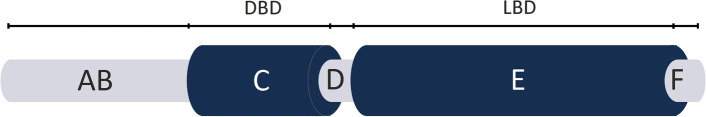
The structural organization of nuclear receptors. Schematic organisation of nuclear receptors including RXRs. NRs exhibit a modular structure with different regions (A–F). Some of the regions correspond to autonomous functional domains: DNA binding domain (DBD) and ligand binding domain (LBD). Contribution of each domain to the nuclear receptor’s activities is different [5]

RXRs have characteristic NR architectures that consist of the AB region, a DNA binding domain (DBD), D region, ligand binding domain (LBD) and F region (Fig. 1) [5]. NRs share a high degree of sequence homology in their DBDs and LBDs but exhibit weak conservancy in the rest of the protein. Many structures of the DBD and LBD of NRs are currently being determined. However, detailed structural information on the AB region, D region and F region is still lacking. They often exhibit properties of intrinsically disordered regions (IDRs) and are characterized by extraordinary structural flexibility and plasticity [6–10]. IDRs have the ability to undergo fast, highly controllable, environment-modulated transitions that are ideally suited for the transient reversible interactions involved in signal transduction and transcriptional regulation [11, 12]. The N-terminal AB region is involved in the modulation of transcriptional activation of target genes in a cell-specific and promoter-dependent manner. It contains a ligand-independent activation function (AF1) region that is recognized and bound by coactivator proteins or other transcription factors [13]. Flexibility of the D region plays an important structural role by permitting rotation of the DBD and LBD [2]. Little is known about the structure and functional role of the F region. Deletion of the F region may perturb NR activity and interaction with coregulators [14].

There are three RXR subtypes, α, β and γ, which are encoded by three distinct genes [1]. The major variation (both in length and amino acid sequence) among them is in the N-terminal AB region (Fig. S1 and Fig. S2). The three RXR subtypes show tissue-specific expression, with partially overlapping functions [15]. RXR subtype β (RXRβ) is widely expressed and can be detected in almost all tissues [1, 16]. RXRβ gene transcripts were also detected in a variety of human tumor cell lines [17]. As RXR expression is rarely lost in human tumors, RXR ligands seem to be promising therapeutic targets for cancer therapy and prevention [18]. Loss-of-function studies performed in mice showed that ∼50% of RXRβ null mutants die in utero [19]. The surviving males were sterile and exhibited testicular defects and abnormal spermatid maturation. The deletion mutants of RXRβ may also lead to abnormal lipid metabolism in Sertoli cells [20], which emphasizes the role of functional interactions of RXRβ with other NRs that control lipid metabolism. Polymorphism of the RXRβ gene has an effect on hepatitis C virus infection [21] and has been identified in schizophrenia patients [22]. Although detailed functional characterization of RXRβ is known, the molecular properties of its AB region, which is critical for several aspects of its action, are still unknown.

An increasing number of reports have shown that some NRs have the ability to liquid–liquid phase separation (LLPS), and the process may be mediated by different regions/domains of NRs. LLPS can be modulated synergistically either by structured domains and/or IDRs of NRs. The structured domains of androgen receptor (AR), PPARγ and glucocorticoid receptor (GR) are the main drivers of LLPS. In particular, the formation of AR and PPARγ liquid condensates is mediated by DBD [23, 24], whereas GR is mediated by LBD [25]. On the other hand, for the estrogen receptor (ER) and RXRγ, AB regions seem to be essential for condensate formation [26, 27]. Additionally, LLPS of RXRγ is driven by hydrophobic interactions, which is quite an unusual feature for IDRs [28].

As the major differences in the subtypes of RXR are in their AB regions, the aim of our study was to reveal the molecular properties of the AB region of human RXRβ (AB_*h*RXRB). We present in silico examinations with comprehensive biochemical and biophysical characteristics of AB_*h*RXRB. Our data demonstrate that AB_*h*RXRB exhibits some properties of IDRs, but it is also characterized by the presence of ordered secondary structure motifs. In particular, AB_*h*RXRB shows the structural and functional characteristics of the coil-like group of intrinsically disordered proteins (IDPs). Moreover, in the presence of external factors such as osmolyte or increased temperature, AB_*h*RXRB reveals a significant propensity to form additional ordered structures. Most importantly, we demonstrate that AB_*h*RXRB, similar to the AB region of RXRγ (AB_*h*RXRG), exhibits potential for LLPS. The unique amino acid composition of AB_*h*RXRB, especially the high content of P amino acid residues and specific response to different environmental factors driving LLPS, govern the distinct phase separation propensity of AB_*h*RXRB. The differences in the amino acid sequence and specific response to factors driving LLPS of a particular AB region of RXR can have a substantial impact on the action of individual subtypes of the receptor.

## Materials and methods

### Chemicals

All buffers were prepared at room temperature. The lysis buffer contained 20 mM Na_2_HPO_4_ and 150 mM NaCl, pH 7.0. Buffer A contained 30 mM Na_2_HPO_4_, 500 mM NaCl, 5% (v/v) glycerol, and 0.5 mM DTT, pH 7.0. Buffer B contained 30 mM Na_2_HPO_4_, 150 mM NaCl, and 5% (v/v) glycerol. Buffer C contained 30 mM Na_2_HPO_4_, 300 mM NaCl, and 5% (v/v) glycerol, pH 7.0. Buffer D contained 30 mM Na_2_HPO_4_, 300 mM NaCl, and 1% (v/v) glycerol, pH 7.0. Buffer E contained 20 mM Tris–HCl, 150 mM NaCl, and 5% (v/v) glycerol, pH 7.0. Buffer IEX_A contained 30 mM Na_2_HPO_4_, 20 mM NaCl, 5% (v/v) glycerol, and 0.5 mM DTT, pH 7.0. Buffer IEX_B contained 30 mM Na_2_HPO_4_, 1 M NaCl, 5% (v/v) glycerol, and 0.5 mM DTT, pH 7.0. PEGs, TMAO, 1,6-hexanediol, Ficoll 70, trehalose, and phenylmethylsulfonyl fluoride (PMSF) were purchased from Sigma–Aldrich, and (NH_4_)_2_SO_4_ was purchased from Carl Roth.

### Preparation of the cDNA construct

The *E*. *coli* strain DH5α (Thermo Fisher Scientific) was used as the host strain for the cloning procedures. The sequence of the full-length human RXRβ (*h*RXRβ) was taken from UniProtKB—P28702. The cDNA of *h*RXRβ was de novo synthetized in GeneArt® (Thermo Fisher Scientific). The gene sequence was optimized using Gene Optimizer software to maximize the expression of the synthetic gene in *E*. *coli*. An optimized sequence was used as the template for the PCR. The following primers were used for amplification: forward: CCGGGGCCATGGGAagctgggcagcacgtccgc; reverse: GCGCGCGCGGCCGCTTAacctgcaccaggaccaccag. The forward primer introduced the *Nco*I restriction site, and the reverse primer introduced the *Not*I restriction site. The capital letters in the sequences represent nucleotides that are added to coding sequences for cloning purposes, whereas the restriction sites are highlighted. The insert was ligated with the pETHSu plasmid in a frame with the sequence that encodes the N-terminal peptide containing the polyhistidine (6xHis) tag and SUMO [29]. The sequence of the obtained construct was verified by DNA sequencing.

### Expression and purification of the AB region of hRXRβ (AB_*h*RXRB)

AB_*h*RXRB (amino acid residues 1–202) was expressed in the *E*. *coli* strain ArcticExpress (DE3) (Agilent). Bacteria harboring the expression vector were grown in ZYM-5052 autoinducing media [30] supplemented with 50 μg/ml kanamycin. The culture was grown at 37 °C and 182 rpm until the optical density (OD_600_) reached 1.0, and then protein was expressed at 16 °C overnight. The cells were harvested by centrifugation at 5000 × g at 4 °C for 15 min. The pellet of bacteria was suspended in ice-cold lysis buffer containing PMSF (0.2 mg/ml) and lysozyme (1 mg/ml) and stored at − 80 °C until use.

The frozen cell suspension supplemented with DNase (20 μg/ml), RNase (20 μg/ml) and a fresh portion of PMSF (0.2 mg/ml) was slowly thawed at 10 °C. Cell lysis was improved by sonication. The cell suspension was sonicated with 5 short 20-s bursts followed by intervals of 30 s for cooling. The resulting suspension was centrifuged at 18 000 × g at 4 °C for 1 h. The soluble fractions containing AB_*h*RXRB supplemented with PMSF (0.2 mg/ml) were purified using immobilized metal affinity chromatography (IMAC). The cell lysate was incubated for 1 h at 10 °C with Talon® Metal Affinity Resin (Clontech), which had been previously equilibrated with lysis buffer. The resin was washed with buffer A and buffer C until the absorbance at 280 nm was lower than 0.1. Then, 0.5 mg SUMO hydrolase (dtUD1) [31] was added to the resin, gently mixed and incubated overnight at 4 °C. The protein was eluted with buffer C, followed by buffer exchange to buffer IEX_A and concentration to a total volume of 500 μl using Amicon Ultracel-4 Centrifugal Filter Units (Merck Millipore) with a cutoff limit of 10 kDa. AB_*h*RXRB was purified to homogeneity using a MonoS 5/50 GL (GE Healthcare Life Sciences) column preequilibrated with buffer IEX_A connected to an ÄKTA Avant system (GE Healthcare Life Sciences). The purification was performed at a 0.5 ml/min flow rate at room temperature. The column was washed with 3 CV of buffer IEX_A followed by a linear gradient of buffer IEX_B for 20 min. Fractions containing the purified AB_*h*RXRB were combined, the buffer was changed to buffer C and the sample was concentrated to 1 mg/ml using Amicon Ultracel-4 Centrifugal Filter Units (Merck Millipore) with a cutoff limit of 10 kDa and then aliquoted into small volumes. The concentration of AB_*h*RXRB was determined spectrophotometrically at 280 nm. The absorption coefficient calculated according to Gill and von Hippel [32] was 0.829 ml/(mg x cm). The samples were stored at − 80 °C. The molecular mass of AB_*h*RXRB was determined in a mass spectrometry laboratory (IBB PAS, Warsaw). All of the experiments presented in this paper were performed using samples obtained from different preparations. The results were reproducible, and we did not observe any variability among the different preparations.

### Sodium dodecyl sulfate–polyacrylamide gel electrophoresis (SDS–PAGE)

The protein samples were analyzed by SDS–PAGE using 4%/15% polyacrylamide gels [33]. Electrophoresis was performed at a constant current of 20 mA/1 mm gel with the Unstained Protein Molecular Weight Marker (Thermo Fisher Scientific). After electrophoresis, the gels were stained with Coomassie Brilliant Blue R-250 [34] and analyzed using Image Lab Software (Bio-Rad).

### Circular dichroism spectroscopy

Circular dichroism (CD) spectra were recorded using a Jasco-815 spectropolarimeter (Jasco Inc) equipped with a Peltier temperature controller (CDF-426S/15). The spectra were collected in a spectral range of 190–260 nm with a scanning speed of 50 nm/min at 20 °C, D.I. T – 2 s and a 1-nm band width. The data pitch was 0.5 nm, and the final spectrum was obtained after averaging three measurements. The spectra were measured using quartz cuvettes with a path length of 1 mm, and the concentration of AB_*h*RXRB was 10 μM (0.2 mg/ml) in buffer B. The measurements in the presence of GdmCl, TFE, TMAO, PEG 8000 and (NH_4_)_2_SO_4_ were performed after 30 min of incubation at room temperature. Temperature-dependent denaturation was monitored by following the changes in ellipticity at 222 nm by increasing the temperature from 20 °C to 70 °C and then decreasing it from 70 °C to 20 °C at a constant rate of 1 °C/min. All the spectra were corrected for the effect of the buffer and converted to molar residual ellipticity units. The molar molecular mass per residue (MRW) for AB_*h*RXRB is 98.99 Da. Evaluation of the secondary structure content was calculated with CDPro deconvolution software using three algorithms: Continll, Selon3 and Cdsstr. IBasis 6 and 7 (SPD42 and SPD48) were used as the reference protein dataset [35].

### Sedimentation velocity analytical ultracentrifugation experiments (SV-AUC)

Sedimentation velocity experiments were performed at 20 °C on a Beckman Coulter ProteomeLab XL-I ultracentrifuge (Beckman Coulter Inc.) in an An-60 Ti rotor. AB_*h*RXRB (400 μL of protein at three concentrations: 0.25 mg/ml, 0.5 mg/mL, or 1.0 mg/mL in buffer D) was loaded in two-channel centerpieces and centrifuged overnight at 50 000 rpm. Detection of the protein concentration was performed using OD measurements at a wavelength of 280 nm. The data were analyzed with SEDFIT software using a continuous size distribution c(s) model to extract the sedimentation coefficient (s) [36, 37]. The partial specific volumes (V_bar_) of AB_*h*RXRB (0.724747 ml/g) and the density (1.0169 g/ml) and dynamic viscosity (0.010627 mPa × s) of the buffer at 20 °C were calculated using SEDNTERP software [38]. Maximum entropy regularization with p = 0.68 was applied. The sedimentation coefficient (s), after correction for the solvent density and viscosity in relation to the density and viscosity of water at 20 °C, was expressed as s_20,w_. The hydrodynamic dimensions of AB_*h*RXRB (R_S_ and M) were calculated by SEDFIT. The quality of the fits was assessed using the RMSD values, residual distributions and residual histograms.

### Limited proteolysis

Purified AB_*h*RXRB (0.5 mg/ml) was digested with proteinase K (A&A Biotechnology) and trypsin (Lonza) at a final concentration of 25 μg/ml, and the endoproteinase Glu-C (V8) (Sigma–Aldrich) was digested using a 1:250 (w/w) substrate-to-protease ratio in the absence or presence of 30% TFE. The control reactions did not contain any enzymes. The proteolysis reaction was conducted at 15 °C in buffer E. After defined time intervals (5, 15 and 60 min), 10 μl samples were taken, mixed with 3 μl SDS loading buffer and heated for 10 min at 95 °C. Cleaved peptides were resolved using 4%/15% SDS–polyacrylamide gels.

### Droplet formation assay

For microscopic and spectroscopic examinations, purified AB_*h*RXRB was concentrated to 400 μM using Amicon Ultracel-4 Centrifugal Filter Units (Merck Millipore) with a cutoff limit of 10 kDa. The protein was mixed in low-binding microtubes (72.706.600; Sarstedt) with phosphate buffer supplemented with the appropriate amount of sodium chloride, PEGs, TMAO, 1,6-hexanodiol, (NH_4_)_2_SO_4_, Fikoll 70 and trehalose. Optical density measurements at 340 nm using a NanoDrop 2000c spectrophotometer (Thermo Scientific) were performed to estimate the turbidity of the protein samples. The droplet formation of the protein samples was monitored using DIC microscopy. Two microliters of the sample was loaded onto glass coverslips, and DIC images were acquired using a Zeiss Axio Observer 7 inverted microscope with a 100 × objective (oil immersion). The images were processed using ZEN 3.0 (ZEN lite).

### Fluorescent labeling of the protein

The AB_*h*RXRB protein was fluorescently labeled using the Atto 488 NHS ester (41,698; Sigma–Aldrich) according to the manufacturer's manual. The calculated degree of labelling was 0.7. To confirm the presence of protein in the droplets, Atto 488-labeled protein was mixed with unlabeled protein, and images were acquired on a Zeiss Axio Observer 7 inverted microscope with a 100 × objective (oil immersion).

### Fluorescence anisotropy measurements

Fluorescence anisotropy measurements were performed on an FP-8300 spectrofluorometer (Jasco) equipped with excitation and emission polarizers and with a Peltier temperature controller (EHC-813). QS High Precision Cell cuvettes (105–251-15–40; Hellma Analytics) were used. All the samples were prepared to a final volume of 60 μl and were monitored at an excitation wavelength of 498 nm and emission wavelength of 520 nm, with 5-nm bandwidths. The changes in fluorescence anisotropy of the AB_*h*RXRB samples in buffer B at different pH values were monitored by increasing the temperature from 5 °C to 50 °C at a constant rate of 1 °C/min. The Atto 488-labeled protein was incubated with unlabeled protein (200 μM) in a 1:100 ratio. Anisotropy (r), including the instrument G factor, was calculated using the Equation $\mathrm{r}=\frac{{I}_{|| }-{I}_{\perp }}{{I}_{|| }+2 \times {I}_{\perp }}$, where ${I}_{|| }$ and ${I}_{\perp }$ are the components of the fluorescence intensity that are parallel and perpendicular to the electric vector of the excitation light, respectively [39].

### Fluorescence spectroscopy

8-anilino-1-naphthalenesulfonic acid (ANS) fluorescence measurements were performed on a Fluorolog-3 spectrofluorometer (Horiba Jobin Yvon Inc.) at 20 °C. The concentration of the ANS stock solutions was calculated using ε_351_ = 6240 M^−1^ × cm^−1^ [40]. The 3 μM AB_*h*RXRB samples containing 20 μM ANS were prepared in buffer E in the presence of 5% PEG 8000 (no LLPS), 0.5 M TMAO (no LLPS), 20% PEG 8000 (LLPS) or 1.5 M TMAO (LLPS). ANS fluorescence was excited at 351 nm, and the emission spectra were recorded in the wavelength range of 400–650 nm. An integration time of 0.5 s and slits with bandwidths of 4 nm were used.

### Sequence analysis

Analysis of the AB_*h*RXRB sequence was performed using bioinformatics tools with the default settings. The ProtParam tool (https://web.expasy.org/protparam/) allows for the computation of various physical and chemical parameters (e.g., molecular mass (M), theoretical pI, extinction coefficient) [41]. The amino acid composition was analyzed using a Composition Profiler (http://www.cprofiler.org) [42]. The linear net charge per residue of AB_*h*RXRB was obtained using the CIDER tool (http://pappulab.wustl.edu/CIDER/analysis/) [43], and hydropathy scores were obtained using ExPASy according to the Kyte & Doolittle method (https://web.expasy.org/protscale/) [41]. The disordered regions were predicted using IUPred2A (short) (https://iupred2a.elte.hu/) [44, 45] and PONDR (VLXT) (http://www.pondr.com) [46] predictors. Protein backbone dynamics were predicted using DynaMine (http://dynamine.ibsquare.be) [47, 48]. Charge-hydropathy analysis and additional disorder prediction were conducted using the PONDR server [46]. To analyze the phase separation propensity of AB_*h*RXRB, four predictors were used: catGRANULE (http://s.tartaglialab.com/update_submission/302811/6360102143) [49], PScore (http://abragam.med.utoronto.ca/~JFKlab/Software/psp.htm) [50], PSPredictor (http://www.pkumdl.cn:8000/PSPredictor/) [51], and FuzDrop (https://fuzdrop.bio.unipd.it/predictor) [52]. The potential sites cleaved by endoproteinase Glu-C (V8), trypsin and proteinase K in the AB_*h*RXRB sequence were predicted using PeptideCutter (https://web.expasy.org/peptide_cutter/) [41]. Alignment of the three human subtypes of RXR sequences was performed using ClustalX 2.1 [53].

## Results

### Expression, purification and in silico analysis of the AB region of hRXRβ (AB_*h*RXRB)

To perform biochemical and biophysical analyses of recombinant AB_*h*RXRB, we elaborated and optimized a protocol for its efficient expression and purification. AB_*h*RXRB was expressed in the *E*. *coli* strain ArcticExpress (DE3) from the pETHSu plasmid encoding the cDNA sequence of the AB region of *h*RXRβ with the N-terminal peptide containing the polyhistidine (6xHis) tag and SUMO. Different thermal conditions (16 °C, 20 °C and 30 °C), media (TB and ZYM-5052 autoinducing media) and IPTG induction times (30 min, 1 h, 2 h, 3 h, 5 h) were evaluated to optimize the overexpression of AB_*h*RXRB (data not shown). After optimization, AB_*h*RXRB was overexpressed at 16 °C overnight in ZYM-5052 autoinducing medium. A temperature higher than 16 °C resulted in more degradant products of AB_*h*RXRB (data not shown). To prevent AB_*h*RXRB degradation, the cell extract was supplemented with PMSF, a serine protease inhibitor. PMSF was added at every working step of the purification procedure until a majority of the contaminant proteins were removed. Additionally, all subsequent steps were carried out on ice or at 4 °C.

**Fig. 2:**
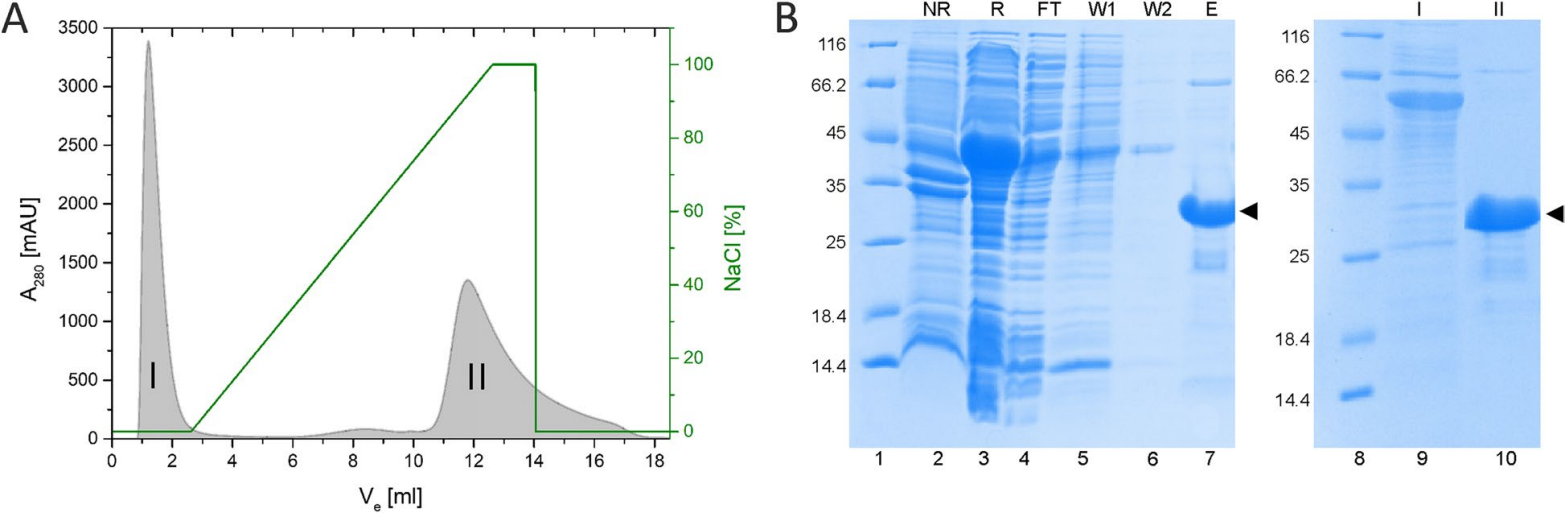
Purification of AB_*h*RXRB. **A** Ion exchange (IEX) chromatography using a MonoS 5/50 GL column. The absorbance was monitored at 280 nm. The green line corresponds to the gradient of the NaCl concentration. Fractions from the II peak, containing pure AB_*h*RXRB, were combined, concentrated and used for further analysis. **B** SDS–PAGE analysis of the expression and purification of AB_*h*RXRB. The gel was stained with Coomassie Brilliant Blue R250. Lanes 1 and 8, molecular mass standard; Lane 2, the fraction of insoluble proteins obtained after cell lysis; Lane 3, the fraction of soluble proteins obtained after cell lysis; Lane 4, the fraction of proteins unbound to Talon® Metal Affinity Resin; Lanes 5 and 6, fraction of proteins eluted with buffers A and C, respectively; Lane 7, the combined fraction after digestion and elution with buffer C; Lane 9, fraction of protein impurities separated during IEX chromatography (peak I); Lane 10, combined fractions of pure AB_*h*RXRB eluted from the MonoS 5/50 GL column (peak II). The arrowhead marks the position of a band corresponding to AB_*h*RXRB

Because the pETHSu plasmid enables protein expression with the 6 × His tag, immobilized metal affinity chromatography (IMAC) using resins containing cobalt ions (Talon® Metal Affinity Resin) was chosen as the first step of the purification procedure. Once bound to the resin, AB_*h*RXRB was washed with a high-salt concentration buffer, which enabled the reduction of contamination without significant protein loss (see Fig. 2B, Lane 5). Next, the ionic strength was lowered, which also enabled the reduction of contaminants (see Fig. 2B, Lane 6) and further digestion with SUMO hydrolase (dtUD1). Preliminary size-exclusion chromatography (SEC) was used as an additional step of purification, but unfortunately, it was not possible to obtain a homogenous protein sample (data not shown) using this technique. Thus, ion exchange chromatography with a MonoS column was used (Fig. 2A). This step turned out to be crucial to separate AB_*h*RXRB (Fig. 2A, II peak) from the remaining contaminants, including the degradation products (Fig. 2A, I peak; Fig. 2B, Lanes 9–10). The described purification procedure yielded up to 14 mg of AB_*h*RXRB from 1 L of ZYM-5052 autoinducing culture medium. Purified AB_*h*RXRB appeared as a single band on the SDS–PAGE gel (Fig. 2B, Lane 10). The molecular mass of AB_*h*RXRB was confirmed by electrospray ionization mass spectrometry (ESI–MS) (19 896 Da) and was in agreement with the molecular mass estimated with the ProtParam tool (19 897 Da). Deconvolution of the mass spectrum showed the presence of a second (minor) species (20 060 Da). The mass difference between the peaks is approximately 163 Da, suggesting glycosylation of AB_*h*RXB [54]. However, additional studies are required to identify and confirm the modification.

Interestingly, SDS–PAGE analysis revealed some abnormal characteristics of AB_*h*RXRB. The molecular mass of AB_*h*RXRB estimated on the basis of mobility in SDS–PAGE was 31.2 kDa. This value is approximately 157% of the theoretical molecular mass value calculated with the ProtParam tool (19.9 kDa) and confirmed in the ESI–MS (19 896 Da). The atypical molecular mobility in the SDS–PAGE experiments is characteristic of intrinsically disordered regions (IDRs), which are known to migrate slower in SDS–PAGE than globular proteins with the same molecular mass. Because of their unique amino acid composition, IDRs do not bind SDS as much as globular proteins. The apparent molecular mass estimated from SDS–PAGE is often 1.2–1.8 times higher than the real mass calculated on the basis of the primary structure [55].

**Fig. 3:**
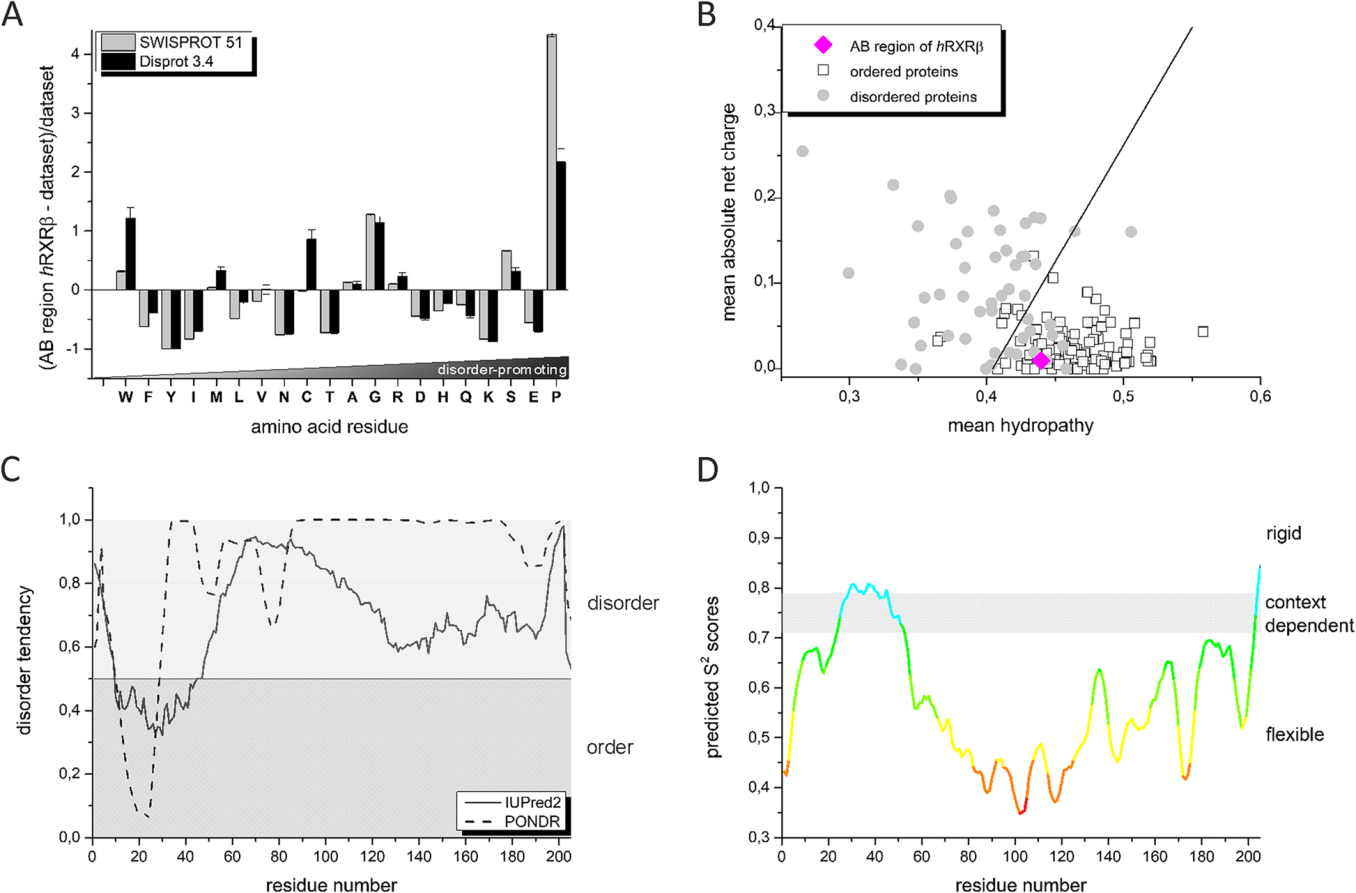
In silico analysis of the AB_*h*RXRB sequence. **A** Analysis of the amino acid composition using the Composition Profiler. The plot illustrates the enrichment (values above zero) and depletion (values below zero) of given amino acid residues relative to the proteins from the SwissProt and Disprot 3.4 (dataset with disordered proteins) databases. The amino acid residues are arranged from the most order-promoting to the most disorder-promoting potential. **B** Uversky plot [46] of the mean hydropathy versus mean absolute net charge of 105 completely ordered proteins (open squares) and 54 completely disordered proteins (gray circles). The solid line represents the border between the ordered and disordered proteins. The magenta diamond corresponds to AB_*h*RXRB. **C.** The prediction of the degree of disorder in the AB_*h*RXRB sequence calculated from the primary structure. Two algorithms were used – IUPRED2 (short) [44, 45] and PONDR (VLXT) [46]. A score above 0.5 indicates a high probability of disorder. **D.** Protein backbone dynamics predicted with DynaMine [47, 48]. An S2 value larger than 0.8 indicates high rigidity of the protein backbone, whereas an S2 value lower than 0.69 indicates high flexibility, which is typical for disordered segments. Values between 0.69 and 0.8 are characteristic of the context-dependent structural organization of polypeptide chains

During the overexpression and purification procedures of AB_*h*RXRB, we observed its high susceptibility to degradation and increased electrophoretic mobility, which might indicate that AB_*h*RXRB exhibits properties of IDRs or intrinsically disordered proteins (IDPs). It has been reported that some AB regions of nuclear receptors (NRs) display characteristic properties of IDRs [6–8, 27]. Intrinsic disorder was suggested to provide benefits to these transcription factors and enable them to carry out their functions. These results inspired us to take a closer look at the molecular properties of AB_*h*RXRB. Analysis of the amino acid composition of AB_*h*RXRB using the Composition Profiler tool [42], which helps to detect the enrichment (values above zero) or depletion (values below zero) of amino acids by their properties, was performed (Fig. 3A). The amino acids are arranged in order of increasing disorder-promoting capacity [56]. The analysis revealed that AB_*h*RXRB is depleted in amino acid residues classified as order-promoting (F and I), and one of them (Y) is completely absent in the sequence. At the same time, the AB_*h*RXRB sequence is rich in amino acid residues characterized as disorder-promoting (G, S, and P). The percentages of P, G and S in AB_*h*RXRB are 25.7%, 15.8% and 11.4%, respectively (Fig. S2). According to the literature, this kind of amino acid distribution is typical of IDRs [57]. However, there were some disorder-promoting residues (D, H, Q, K, and E) that were clearly underrepresented in the AB_*h*RXRB sequence. Additionally, the AB_*h*RXRB sequence indicated considerable enrichment of W, an order-promoting amino acid residue. In AB_*h*RXRB, there are three W residues. Thus, analysis of the amino acid composition of AB_*h*RXRB revealed the dual character of its sequence. In particular, AB_*h*RXRB possesses a mixture of amino acid residues, which is characteristic of disordered sequences as well as ordered proteins. The charge-hydropathy plot [58] classified AB_*h*RXRB into the group of ordered proteins (Fig. 3B). The position occupied by AB_*h*RXRB is quite ambiguous to interpret, as it emerges within the region occupied by both ordered and disordered proteins.

To estimate the occurrence of the putative intrinsic disorder propensity of AB_*h*RXRB, in silico analysis using different disorder predictors was performed. Since the results of most of the predictors were compatible, we decided to show the data obtained for IUPRED2 [44, 45] and PONDR [46] predictors (Fig. 3C). More than 80% of the analyzed sequence seems to be disordered. Only the sequence spanning the 40 amino acid residues in the N-terminus of AB_*h*RXRB was predicted as a potential ordered region. Additional in silico analysis conducted using DynaMine algorithms (Fig. 3D), a tool that is designated for the prediction of protein backbone dynamics [47], was in agreement with the results obtained using disorder predictors. These data clearly indicate that AB_*h*RXRB is highly disordered.

Altogether, the unusual properties of AB_*h*RXRB that were observed, including the differences between the calculated molecular mass value and SDS-PAGE analysis results, the characteristic amino acid sequence composition and the predicted presence of disordered regions suggest that AB_*h*RXRB exhibits properties of an IDR.

### Hydrodynamic properties of AB_*h*RXRB

**Fig. 4:**
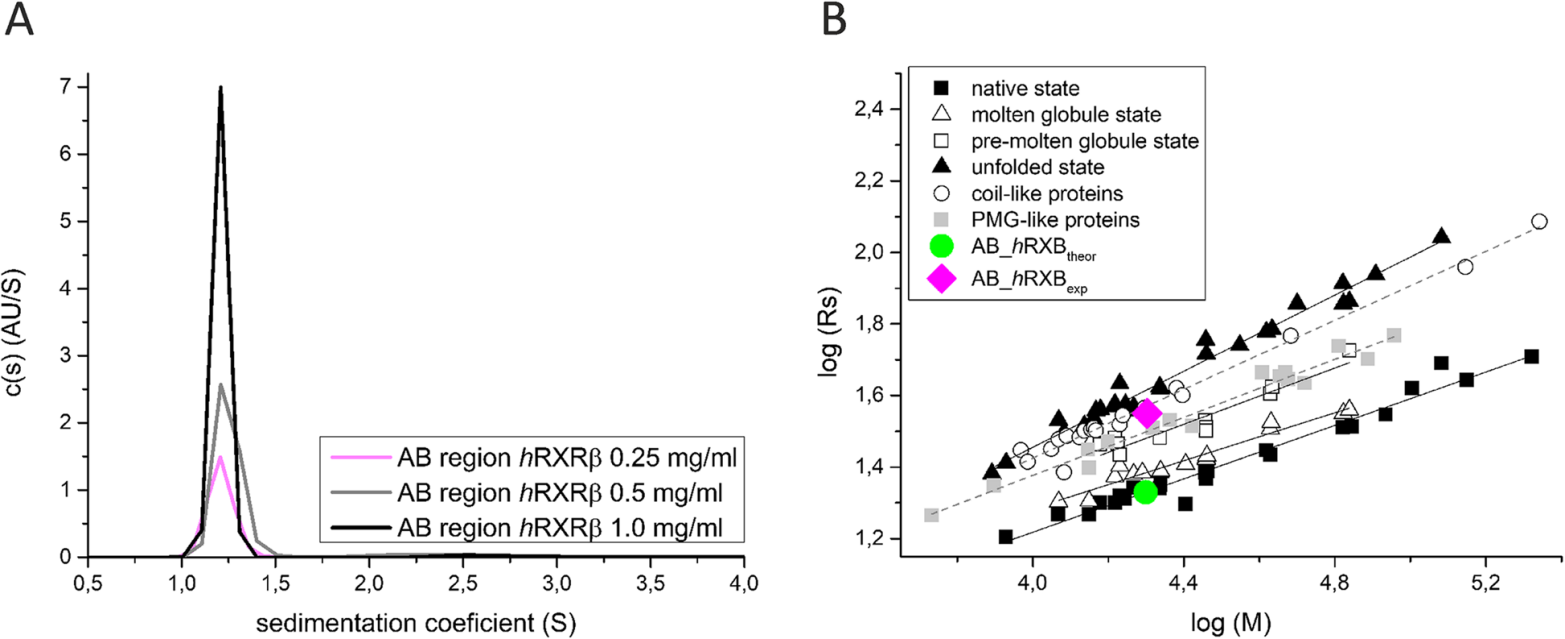
Hydrodynamic properties of AB_*h*RXRB. **A** Sedimentation velocity analysis (SV-AUC). Superposition of the sedimentation coefficient c(s) distributions for three different concentrations of AB_*h*RXRB: 0.25 mg/ml (magenta line), 0.5 mg/ml (gray line) and 1.0 mg/ml (black line). **B** The plot presents the logarithmic values of the hydrodynamic radii (R_s_) versus the logarithmic values of the molecular mass (M) for proteins in the native state (black squares), molten globule state (white triangle), premolten globule state (white squares), unfolded state (black triangle), coil-like proteins (white circle) and PMG-like proteins (gray squares). The data for globular proteins were taken from [60], and the data for IDPs are from [58]. The green circle corresponds to the R_s_ of AB_*h*RXRB calculated from the equation log (R_s_^native^) = (0.373 ± 0.014) × log (M)—0.274 ± 0.064, assuming it is a globular protein (AB_*h*RXRB_theor_). The magenta diamond corresponds to the R_s_ determined for AB_*h*RXRB after AUC analysis (AB_*h*RXRB_exp_)

To determine the hydrodynamic properties of AB_*h*RXRB, sedimentation velocity analytical ultracentrifugation (SV-AUC) experiments were applied. The results are summarized in Table 1. The sedimentation coefficient distributions calculated from the SV-AUC data indicate that one major species with a sedimentation coefficient (s_20. W_) of approximately 1.3 S was observed at three different concentrations (0.25 mg/ml, 0.5 mg/ml and 1 mg/ml) (Fig. 4A); data analysis for all three concentrations yielded a good fit, with RMSD values of 0.007598, 0.009368 and 0.010791, respectively (Table 1 and Fig. S3). No significant dependence of the sedimentation coefficient (s_20. W_) on the protein concentration was observed. The estimated molecular mass (19 144 Da, 21 507 Da and 19 426 Da) was close to the theoretical value (19 897 Da), and the c(s) distribution obtained for different protein concentrations overlapped (Fig. 4A), indicating that AB_*h*RXRB is a monomer. The obtained Stokes radii (R_s_) for the different concentrations of AB_*h*RXRB were 34.5 Å, 37.4 Å and 35.7 Å. These values are considerably higher than the 21.4 Å value calculated from the sequence data assuming that AB_*h*RXRB is a globular protein (Fig. 4B). The remarkably larger experimental R_s_ in comparison to the theoretical value places AB_*h*RXRB on the log (R_s_) versus log (M) plot in the area for IDPs with coil-like properties (Fig. 4B). This classification is in agreement with the value of the frictional ratio (f/f_0_) obtained in the SV-AUC experiment. Globular proteins are characterized by values of approximately 1.2–1.25, whereas IDPs in which the molecules have extended shapes are characterized by much larger values of f/f_0_ [59]. Furthermore, the f/f_0_ ratios are typically 2.1 for the 20 kDa and 3.0 for the 200 kDa coil-like IDPs and 1.75 for the 20 kDa and 2.05 for the 200 kDa PMG-like IDPs. The calculated f/f_0_ ratio for AB_*h*RXRB is 2.0 (Table 1), which indicates an extended conformation and classification of AB_*h*RXRB as a coil-like IDP.

**Table 1:** SV-AUC data obtained for AB_*h*RXRB

c [mg/ml]	RMSD	f/f_0_	M [Da]	R_s_ [Å]	s_20.w_ [S]
0.25	0.007598	1.955099	19 144	34.5	1.349
0.5	0.009368	2.037723	21 507	37.4	1.398
1.0	0.010791	2.014038	19 426	35.7	1.322

In summary, the data presented above indicate that AB_*h*RXRB exhibits a highly extended conformation with properties characteristic of coil-like IDPs. Moreover, AB_*h*RXRB exists as a monomer and does not exhibit a propensity for oligomerization.

### Secondary structure analysis of AB_*h*RXRB

**Fig. 5:**
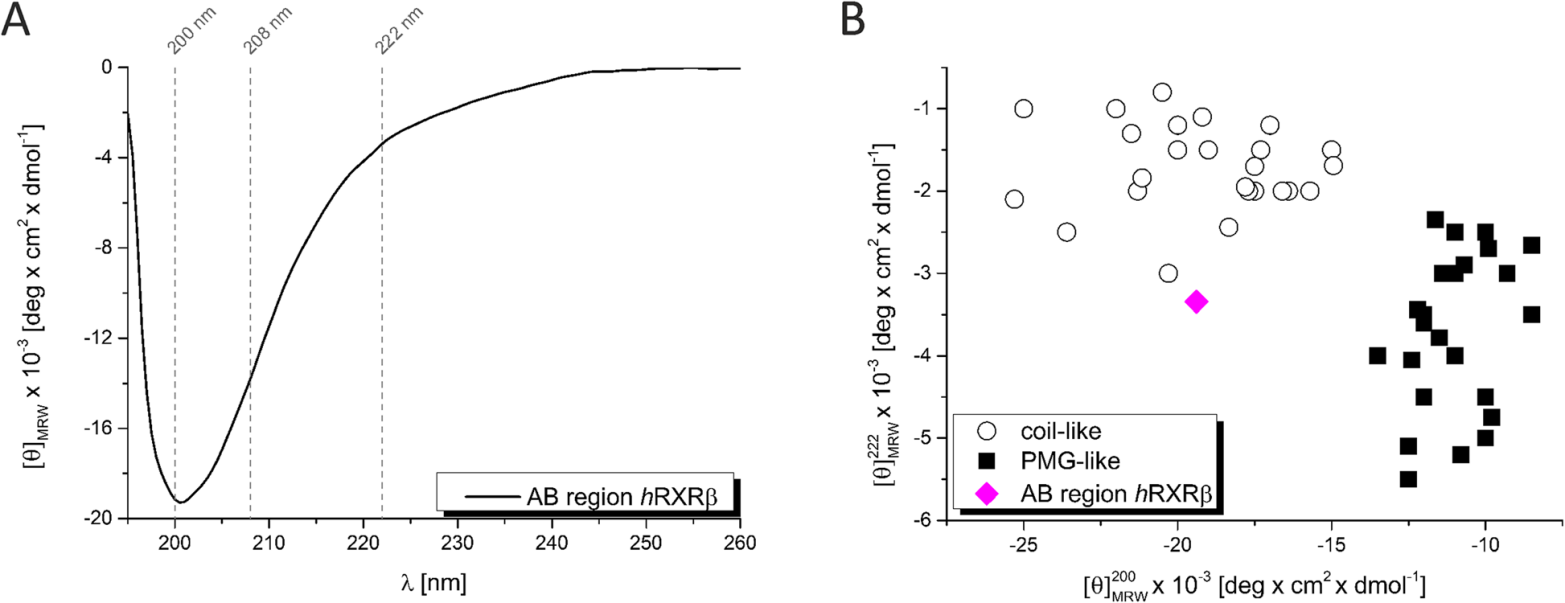
CD spectrum of AB_*h*RXRB. **A** The far-UV spectrum of AB_*h*RXRB. The protein concentration was 10 μM (0.2 mg/ml). The spectrum was recorded at 20 °C. **B** Classification of the conformational states of AB_*h*RXRB. The double wavelength plot showing [θ]_222_ versus [θ]_200_ of the PMG-like proteins (black squares) and coil-like proteins (circles). The data were taken from [62]. AB_*h*RXRB is marked with a magenta diamond

Circular dichroism (CD) is a method for evaluating the secondary structure and folding properties of proteins. Based on the shape of the CD spectra in far-UV (240 − 180 nm), it is possible to determine the type of secondary structure present in the analyzed sample [61]. The obtained far-UV CD spectrum of AB_*h*RXRB is typical for proteins containing disordered regions (Fig. 5A). It is characterized by a deep minimum at 200 nm and a lack of distinct minima at 208 nm and 222 nm. To quantitatively evaluate the content of the secondary structure, the CD spectrum was analyzed by CDPro deconvolution software with IBasis 6 and 7 as the reference protein dataset [35]. Three algorithms were used: Continll, Cdsstr and Selcon3 [35]. Deconvolution of the spectrum revealed that 54.5% ± 1.7 of the sequence is unordered (Table 2). AB_*h*RXRB also possesses a substantial amount of ordered secondary structure motifs, which could be related to the unique amino acid composition of the sequence (Fig. 3A) and might explain the position of AB_*h*RXRB on the charge-hydrophathy plot (Fig. 3B). The prevalent type of ordered structure is the β-strand (18.2% ± 2.0). The α-helical structure was estimated to be 13.9% ± 2.0 and turns comprise 16.7% ± 1.1 (Table 2).

**Table 2:** Estimation of the secondary structure content in AB_*h*RXRB based on CD spectra

Agent	α-helix (%)			β-strand (%)			Turns (%)	Unordered (%)
	Regular	Distorted	Total	Regular	Distorted	Total		
-	7.5 ± 1.3	6.4 ± 0.7	13.9 ± 2.0	12 ± 1.6	6.2 ± 0.4	18.2 ± 2.0	16.7 ± 1.1	54.5 ± 1.7
30% TFE	10.3 ± 0.9	10.1 ± 1.1	20.4 ± 2.0	7.6 ± 1.3	5.1 ± 0.7	12.7 ± 2.0	17.5 ± 1.3	49.0 ± 3.5
50% TFE	13.1 ± 1.6	17.7 ± 1.4	30.7 ± 3.0	10.9 ± 2.1	9.4 ± 1.2	20.3 ± 3.3	22.5 ± 2.3	23.5 ± 3.1
70 °C	10.4 ± 1.6	10.8 ± 0.2	21.2 ± 1.8	14.3 ± 1.8	13.0 ± 2.0	27.2 ± 3.8	23.3 ± 2.2	32.4 ± 3.3

A double-wavelength plot [θ]_222_ versus [θ]_200_ is used to classify IDPs into two structurally different groups: coil-like and PMG-like [62]. Proteins from the first group have an extended conformation that is typical of random coils, whereas the second group consists of proteins that are more compact, although their structure is still less dense than native or molten globule proteins. According to data obtained from the CD spectrum, AB_*h*RXRB belongs to the group of proteins that are coil-like rather than PMG-like (Fig. 5B).

Data calculated from the CD spectra of AB_*h*RXRB support the observations obtained from the hydrodynamic property analyses comprising AUC experiments showing that AB_*h*RXRB exhibits properties of coil-like IDPs.

### Folding and unfolding properties of AB_*h*RXRB

The sensitivity of the protein to differential environmental factors might provide information about its conformation and compactness [63]. Although IDPs/IDRs lack the ability to fold into a stable three-dimensional structure, the formation of ordered secondary structural elements, e.g., α-helices, can be induced by different factors [64, 65]. The CD in far-UV in the presence of guanidinium chloride (GdmCl), osmolyte and at various temperatures were recorded to test whether AB_*h*RXRB exhibits properties characteristic of IDPs/IDRs and whether it can adopt a more ordered structure in the presence of these factors.

**Fig. 6:**
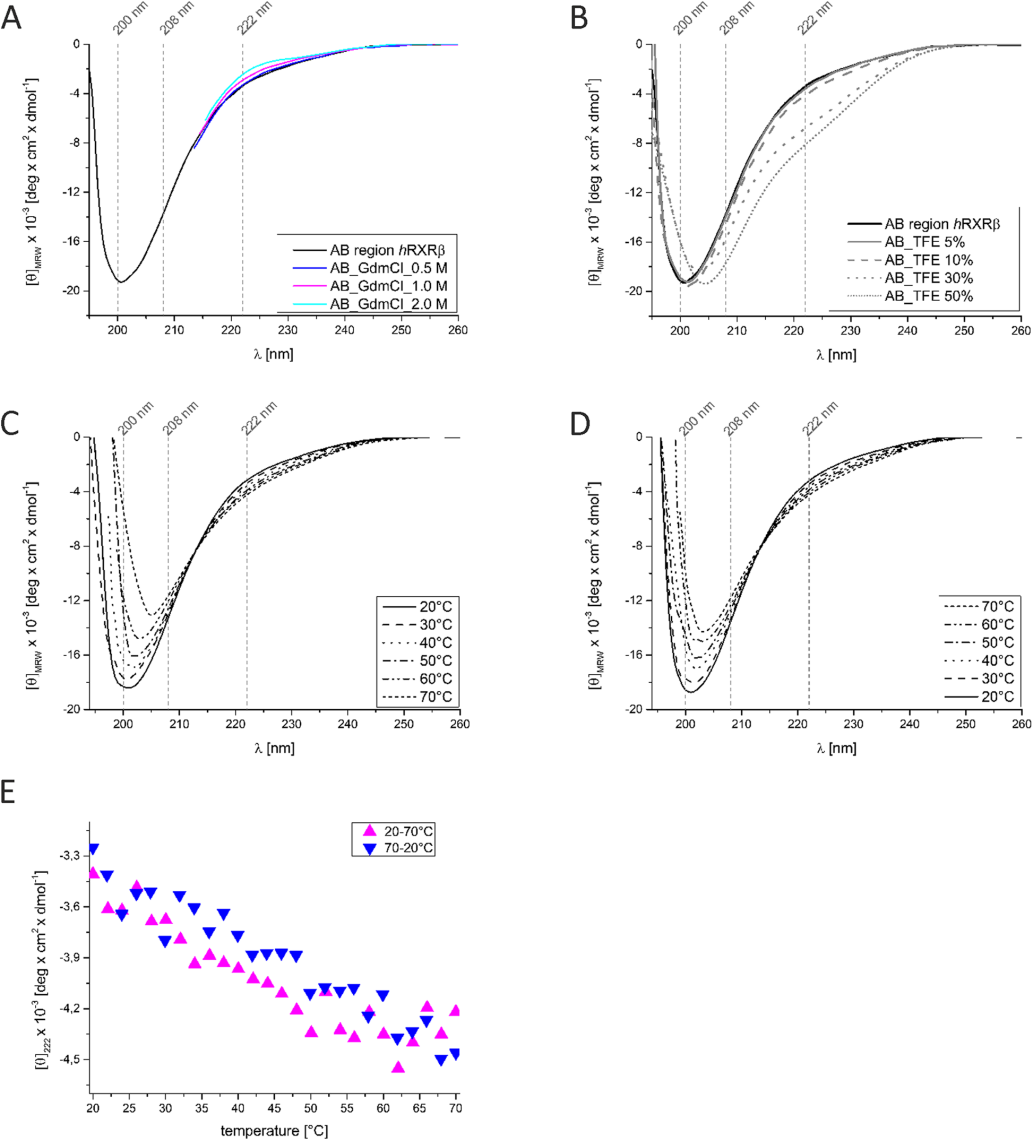
The effects of GdmCl, TFE and temperature on the content of the secondary structure of AB_*h*RXRB. **A** Far-UV CD spectra of AB_*h*RXRB in the absence (black) and presence of increasing concentrations of GdmCl (0.5 M – blue, 1.0 M—magenta and 2.0 M—cyan). **B** Far-UV CD spectra of AB_*h*RXRB in the absence (black) and presence of increasing concentrations of TFE. **C** Temperature-induced secondary structure formation in AB_*h*RXRB. Representative CD spectra of AB_*h*RXRB measured at temperatures from 20 °C to 70 °C and **D.** from 70 °C to 20 °C. **E** Temperature-induced changes in the CD spectrum ([θ]_222_ of AB_*h*RXRB versus temperature) measured at temperatures from 20 °C to 70 °C (magenta triangles) and from 70 °C to 20 °C (blue triangles) at 1 °C intervals

To gain experimental information about the occurrence of the ordered secondary structure, CD measurements were carried out in the presence of the chemical denaturant GdmCl (Fig. 6A). The AB_*h*RXRB samples were incubated 1 h before the measurements with an appropriate concentration of GdmCl (0.5 M, 1.0 M and 2.0 M). It was impossible to collect CD data at approximately 200 nm in the presence of GdmCl because of high values of the HT voltage (above 700 V) as well as the quantitative analysis of the whole spectra in the presence of GdmCl with CDPro software. However, changes in the ellipticity at 222 nm with increasing concentrations of GdmCl were observed. When GdmCl concentrations were equal to or below 0.5 M, relatively poor changes in the CD spectrum were observed. However, at higher concentrations of the denaturant (1 M and 2 M), the ellipticity at 222 nm increased, reflecting the loss of the residual ordered structure.

IDPs can form a more ordered structure upon binding to a specific partner (e.g., another protein or a low molecular weight ligand). Osmolytes have become important factors that mimic the in vivo conditions under which IDPs/IDRs interact with target molecules and adopt a more ordered structure [66]. To test the structure-forming potential of AB_*h*RXRB, we used 2,2,2-trifluoroethanol (TFE), the osmolyte that contributes to the development of the α-helix structure [67, 68]. Figure 6B shows the impact of increasing TFE concentrations on the far-UV CD spectrum of AB_*h*RXRB. At lower concentrations of TFE (5% and 10%), the shapes of the CD spectra were almost the same as those in the absence of osmolytes (Fig. 6B). At 20–25% concentrations of TFE, precipitation of AB_*h*RXRB was observed (data not shown). It was reported that TFE can also induce the formation of β-structure-enriched oligomers, which aggregate and form insoluble precipitates [69]. At higher concentrations of TFE (30% and 50%), substantial changes in the CD spectra were observed, which correspond to the induction of an ordered structure accompanied by a loss in the disordered sequence (Fig. 6B and Table 2). The disappearance of the minimum at 200 nm and the formation of two deep ellipticity minima at 208 nm and 222 nm were accompanied by the formation of the secondary structure in AB_*h*RXRB. The quantitative analysis of the spectra in the presence of TFE obtained using CDPro software indicated that the helical content increased from 13.9% ± 2.0 in the absence of TFE to 30.7% ± 3.0 in the presence of 50% TFE (Table 2). The β-strand content first decreased from 18.2% ± 2.0 in the absence of TFE to 12.7% ± 2.0 in the presence of 30% TFE and then increased to 20.3% ± 3.3 in the presence of 50% TFE. The content of the unordered sequence also decreased from 54.5 ± 1.7 in the absence of TFE to 23.5 ± 3.1 in the presence of 50% TFE. The content of turns increased from 16.7 ± 1.1 in the absence of TFE to 22.5 ± 2.3 in the presence of 50% TFE. Thus, AB_*h*RXRB underwent a conformational change in the presence of TFE, giving rise to an ordered structure with a prevalence of α-helix.

The analysis of the temperature effect on the structural properties of IDPs reveals that they exhibit a so-called turned out response to heat, where increasing temperatures induce the formation of a secondary structure [63]. AB_*h*RXRB displays such properties, as illustrated in Fig. 6C and D, which show the temperature-induced changes in the far-UV CD spectrum of AB_*h*RXRB and the temperature-dependence of the ellipticity at 222 nm (Fig. 6E). As discussed above, at low temperatures, the CD spectrum of AB_*h*RXRB is typical of an unfolded polypeptide chain. As the temperature was increased, the shape of the spectrum changed, reflecting the temperature-induced formation of ordered secondary structure motifs (Fig. 6C). Additionally, as the temperature increased (from 20 °C to 70 °C), the values of [θ]_222_ gradually decreased (Fig. 6E; magenta triangles). The changes in the [θ]_222_ versus temperature plot are linear, suggesting noncooperative folding. The structural heating-induced changes in AB_*h*RXRB were completely reversible (Fig. 6E; blue triangles). The CD spectrum recorded at 20 °C for AB_*h*RXRB that was previously heated to 70 °C (Fig. 6C) and then cooled to 20 °C (Fig. 6D) perfectly matched the CD spectrum of the sample before denaturation (Fig. 6C). Quantitative analysis of the denatured spectrum at 70 °C was performed with CDPro software (Table 2). Compared to data obtained for AB_*h*RXRB at 20 °C, the results show substantial changes in the content of α-helix (from 13.9% ± 2.0 at 20 °C to 21.2% ± 1.8 at 70 °C) and unordered sequence (from 54.5% ± 1.7 at 20 °C to 32.4% ± 3.3 at 70 °C). Moreover, changes were also observed in the content of β-strands (from 18.2% ± 2.0 at 20 °C to 27.2% ± 3.8 at 70 °C) and turns (from 16.7% ± 1.1 at 20 °C to 23.3% ± 2.2 at 70 °C). The analyses presented above clearly demonstrate that AB_*h*RXRB exhibits properties characteristic of IDPs/IDRs in response to increased temperature.

Altogether, the above results indicate that AB_*h*RXRB is an IDR that has a flexible and dynamic structure. The AB_*h*RXRB structure is very sensitive to external factors and can be easily modulated by them, leading to the formation of ordered secondary structure motifs.

### Effect of TFE on the digestion resistance of AB_*h*RXRB

**Fig. 7:**
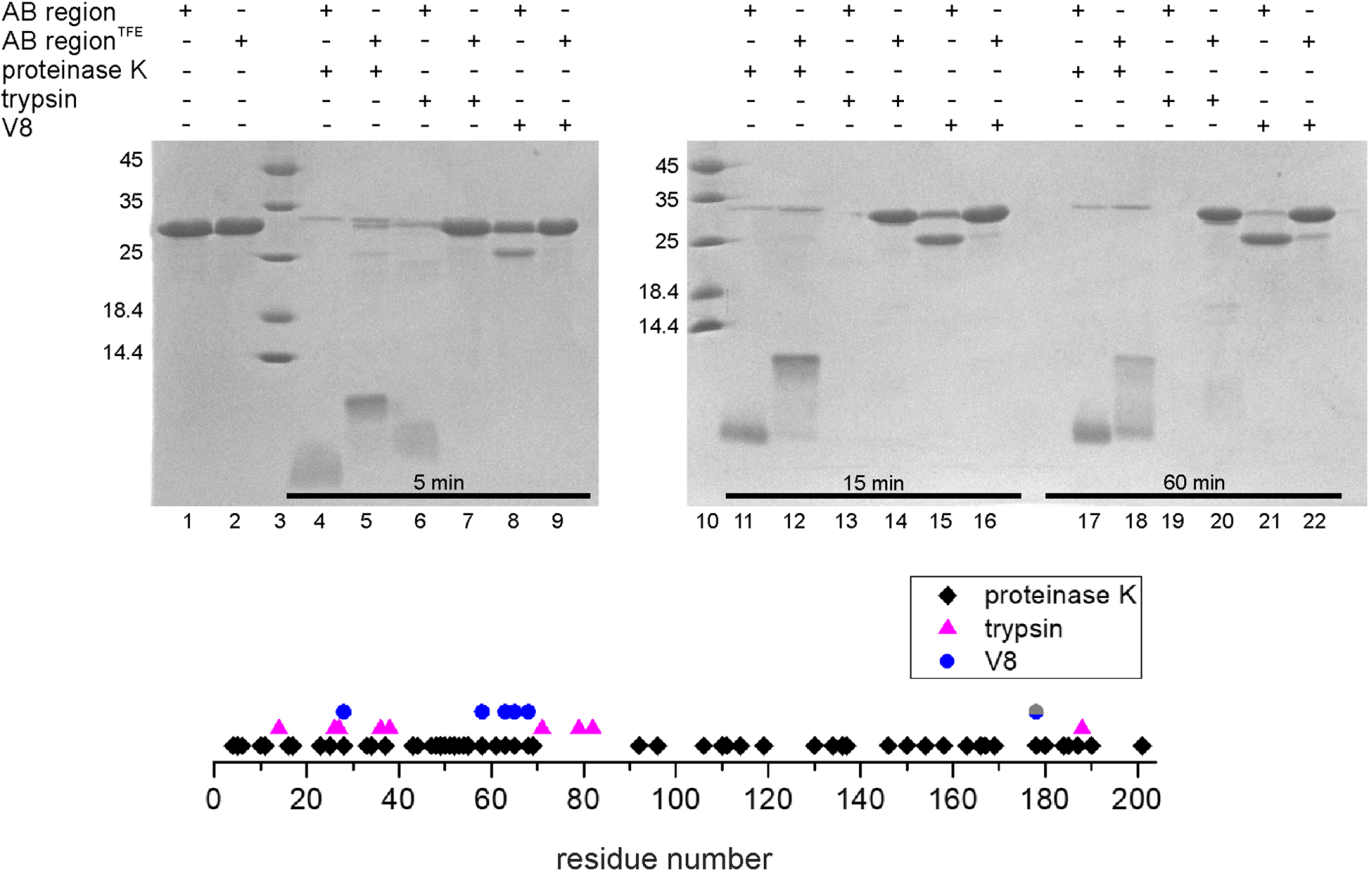
Effects of TFE on the proteolysis of AB_*h*RXRB by proteinase K, trypsin and endoproteinase Glu-C (V8). Purified AB_*h*RXRB alone or in the presence of 30% TFE was digested with proteinase K (Lanes 4–5; 11–12 and 17–18), trypsin (Lanes 6–7; 13–14 and 19–20), or endoproteinase Glu-C (V8) (Lanes 8–9; 15–16 and 21–22). Proteolysis was conducted at 15 °C for an hour. The control samples were the intact AB_*h*RXRB without TFE (Lane 1) and that in the presence of 30% TFE (Lane 2). The molecular weight marker is shown on the left in kDa (Lanes 3 and 10). Aliquots were taken at 5 min (Lanes 4–9), 15 min (Lanes 11–16) and after 60 min (Lanes 17–22) after the addition of enzymes. The scheme in the lower panel depicts the putative protease cleavage sites of AB_*h*RXRB

Flexible protein regions are known to be easily accessible targets for protases. The addition of binding partners or osmolytes often leads to a protein conformation that is less solvent-exposed and thus less sensitive to protease degradation [70]. To examine changes in AB_*h*RXRB conformation, three proteases (proteinase K, trypsin and endoproteinase Glu-C (V8)) were chosen to test the proteolytic cleavage of AB_*h*RXRB in the absence and presence of 30% TFE. Moreover, we wanted to verify whether and to what extent the putative cleavage sites are accessible to proteases. The concentration of the osmolyte was chosen based on the CD data (Fig. 6B) to select the concentration at which substantial changes in the CD spectrum were observed compared to the lower concentration of TFE. AB_*h*RXRB possesses 56 putative cleavage sites for proteinase K, 9 for trypsin, and 6 for V8 (Fig. 7). The proteolytic susceptibility of AB_*h*RXRB to proteinase K, trypsin and V8 in the absence and presence of 30% TFE was analyzed by SDS–PAGE (Fig. 7). AB_*h*RXRB alone or in the presence of 30% TFE was stable during 1 h of incubation (Fig. 7, Lanes 1–2). AB_*h*RXRB in the absence of osmolyte appeared to be very sensitive to proteinase K digestion (Fig. 7; Lanes 4, 11, 17), trypsin (Fig. 7; Lanes 6, 13 and 19) and V8 (Fig. 7; Lanes 8, 15 and 21). Moreover, we observed the difference in susceptibility of AB*h*RXRB sequence to chosen proteases in the absence of TFE. Although AB_*h*RXRB possesses 56 putative cleavage sites for proteinase K, we still observed cleavage products with a relatively high molecular mass (Fig. 7; Lane 11), comparing to reaction in the presence of trypsin (9 putative cleavage sites) where after 15 min the digestion products were not detected (Fig. 7; Lane 13). On the other hand, in the presence of 30% TFE we observed protection against proteinase K (Fig. 7; Lanes 5, 12 and 18) trypsin (Fig. 7; Lanes 7, 14 and 20) and V8 (Fig. 7; Lanes 9, 16 and 22) action when compared to AB_*h*RXRB in the absence of osmolyte. The results showed that in the presence of TFE, AB*h*RXRB sequence was less solvent-exposed and thus less sensitive to protease degradation. Better protection was observed for trypsin and V8, as the substrate sequence possesses only 9 and 6 potential cleavage sites, respectively. This restricted digestion of AB_*h*RXRB in the presence of 30% TFE along with the CD data confirm that AB_*h*RXRB adopts a folded conformation in the presence of this osmolyte.

### Phase separation propensity of AB_*h*RXRB

**Fig. 8:**
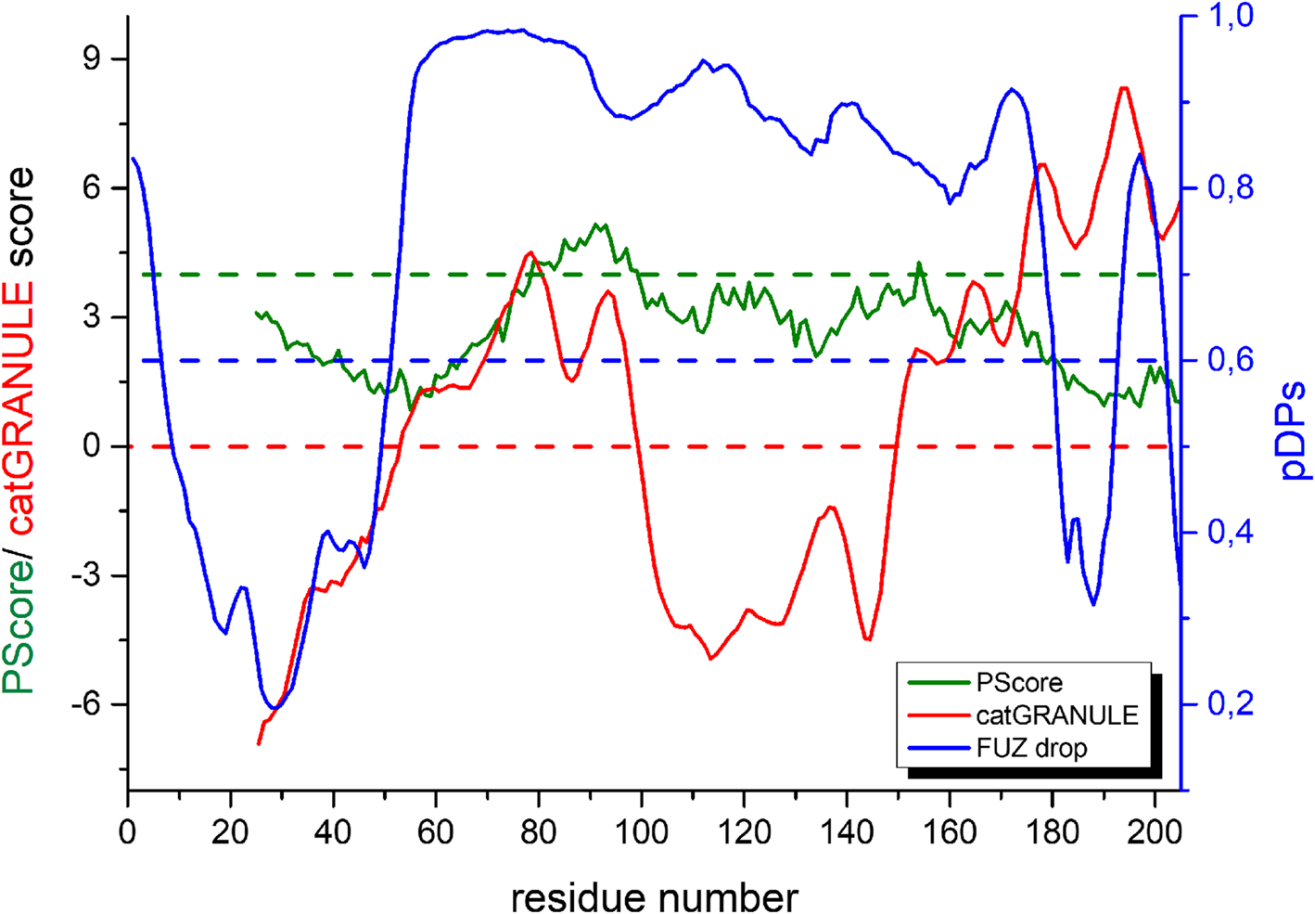
Prediction of the phase separation propensity of the AB_*h*RXRB sequence. Graph illustrating catGRANULE (red line), PScore (green line) and FuzDrop (blue line) analysis results for the AB_*h*RXRB sequence. Values above 0 (red dashed line) and above 4 (green dashed lines) indicate a putative tendency of protein fragments for driving LLPS predicted by the catGRANULE and PScore predictors, respectively, whereas values (pDPs – droplet-promoting probabilities) above 0.6 (blue dashed line) indicate a probable tendency predicted by FuzDrop

The major differences in the sequence of the subtypes of *h*RXR are located in the AB region (Fig. S1 and Fig. S2). The AB region of NRs contributes significantly to the cell and tissue specificity of the action of NRs. It was previously shown that the AB region of *h*RXRγ (AB_*h*RXRG) has a propensity for liquid–liquid phase separation (LLPS) [27]. Thus, we decided to investigate whether AB_*h*RXRB has phase separation propensity, as it might be an important element defining the differences in *h*RXR action. The sequence of AB_*h*RXRB was subjected to three LLPS predictors: catGRANULE [49], PScore [50], and FuzDrop [52] (Fig. 8). Each of these algorithms uses different criteria (e.g., sequence composition, disorder propensities, nucleic acid-binding properties, propensity for long-range planar π-π contacts) and gives score values indicating the protein’s propensity to LLPS. The catGRANULE, PScore and FuzDrop analyses indicated two (54–97 and 150–202 amino acid residues), one (79–99 amino acid residues), and three (1–17, 53–178 and 194–202 amino acid residues) fragments of the AB_*h*RXRB sequence with positive residue score values, respectively. Additionally, each of these tools gave positive total propensity scores to AB_*h*RXRB (Table 3). The FuzDrop analysis also showed the presence of probable droplet-promoting regions (data not shown) in the AB_*h*RXRB sequence; along with the high total propensity score (values above 0.6), this led to the classification of AB_*h*RXRB as a droplet-driver protein. In summary, analyses using different predictors clearly demonstrate that AB_*h*RXRB has a propensity to induce LLPS.

**Table 3:** Total propensity scores for the LLPS of the AB_*h*RXRB sequence

Predictor	Total propensity score
catGRANULE [49]	0.914
PScore [50]	4.30
FuzDrop [52]	0.9984
PSPredictor [51]	0.9913

**Fig. 9:**
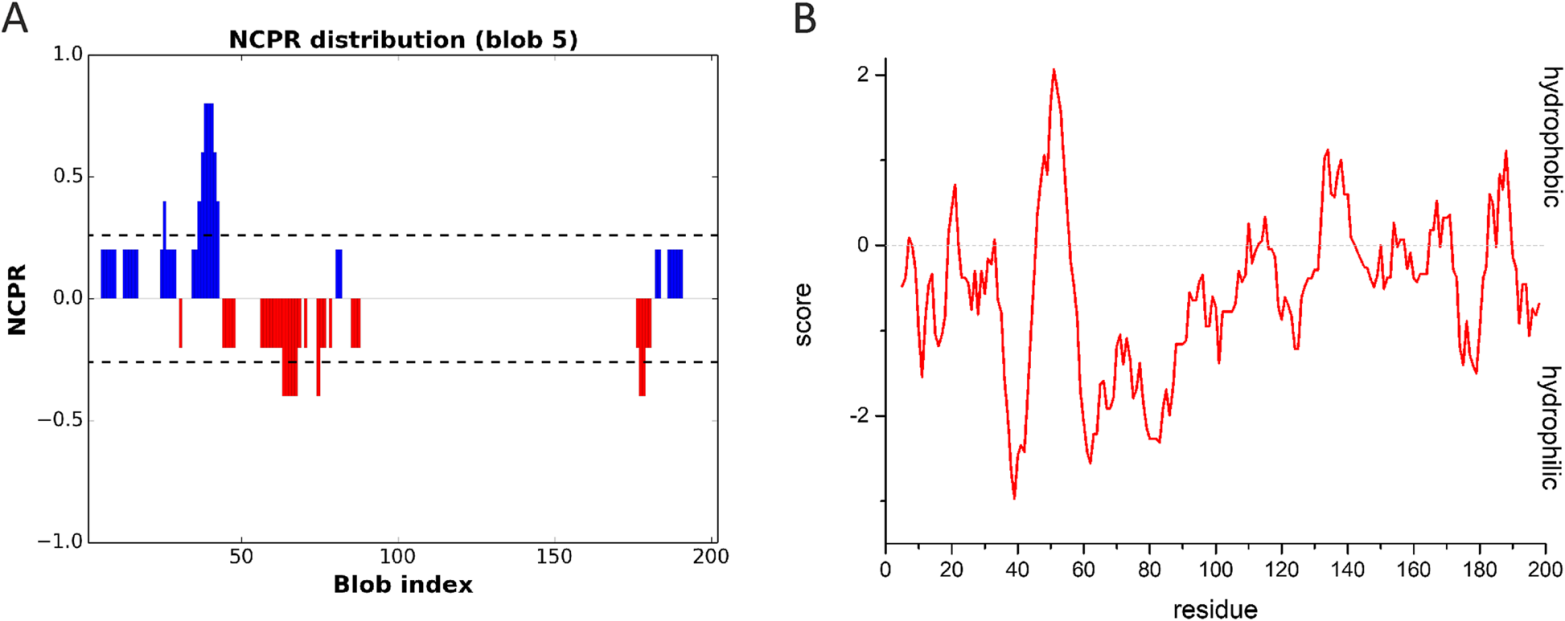
Primary sequence analysis of AB_*h*RXRB. **A** Linear net charge per residue (NCPR) plot of AB_*h*RXRB using the CIDER tool [43]. Positive net charges (blue) and negative net charges (red) are represented. **B** The Kyte & Doolittle plot [41] estimates the hydropathy scores of the AB_*h*RXRB residues. Residues with positive scores are predicted to be hydrophobic, while residues with negative scores are predicted to be hydrophilic

Different types of transient interactions may underlie and stabilize liquid condensates, including electrostatic, dipole–dipole, π − π, cation − π, hydrophobic, and hydrogen bonding interactions [71, 72]. The degree to which side chain and backbone interactions contribute to LLPS depends on the amino acid composition and the overall sequence patterns of the protein [73]. We asked whether the primary structure of AB_*h*RXRB might indicate a mechanism for the formation of liquid condensates. The AB_*h*RXRB sequence is abundant in P (25.7%), G (15.8%) and S (11.4%) amino acid residues (Fig. S2). It also contains a motif consisting of seven A amino acid residues. Using the Classification of Intrinsically Disordered Ensemble Regions (CIDER) tool [73], we obtained a linear net charge per residue (NCPR) plot (Fig. 9A), which indicates fractions of negatively and positively charged residues located mainly in the N-terminus of AB_*h*RXRB, followed by a hydrophobic patch. The calculated values place AB_*h*RXRB on the Das-Pappus phase diagram in the group of weak polyampholytes (Fig. S4). A Kyte & Doolittle plot for AB_*h*RXRB, which represents the average hydropathy along the amino acid sequence, showed that the sequence is hydrophilic rather than hydrophobic (Fig. 9B). The AB_*h*RXRB sequence has a high content of P amino acid residues, for which the individual value on the hydropathy/Kyte & Doolittle scale is – 1.600. Despite the hydrophobic side chain, the P amino acid residue is very hydrophilic. Altogether, the above results indicate that the first-order structure of AB_*h*RXRB is a combination of amino acid residues, which can indicate that a variety of molecular interactions might contribute to the formation of protein liquid condensates.

As in silico analysis of the AB_*h*RXRB sequence did not provide clear information about the types of interactions driving LLPS, we experimentally determined the phase separation propensity of AB_*h*RXRB. First, to probe the contribution of electrostatic interactions, we analyzed a solution containing AB_*h*RXRB at a wide range of protein concentrations and ionic strengths (NaCl concentrations) (data not shown) utilizing both turbidity measurements (optical density at 340 nm) and differential interference contrast (DIC) microscopy. However, AB_*h*RXRB did not undergo LLPS in response to changing NaCl concentration. This result suggests that electrostatic interactions do not contribute to the LLPS of AB_*h*RXRB.

**Fig. 10:**
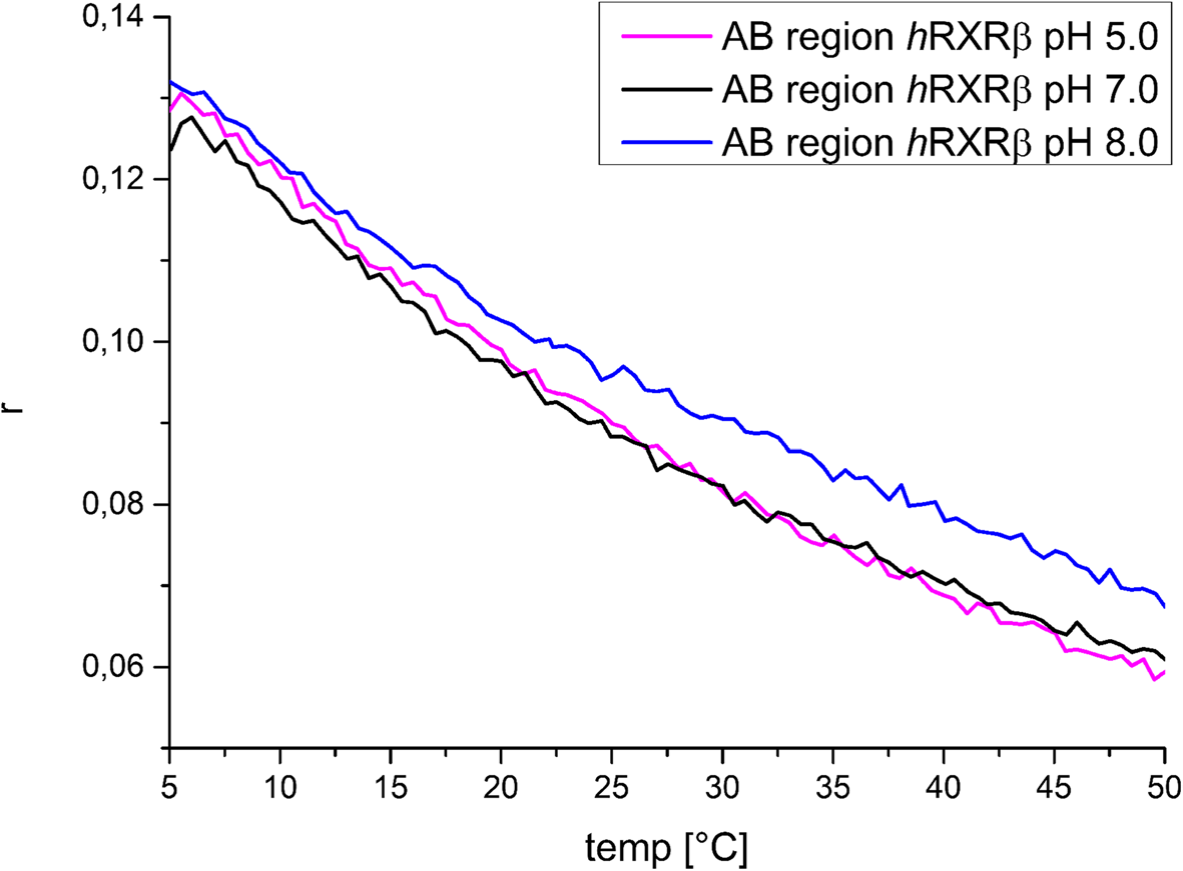
The effects of temperature and pH on the fluorescence anisotropy of AB_*h*RXRB. Temperature-induced changes in the fluorescence anisotropy (r) of Atto 488-labeled AB_*h*RXRB measured at temperatures of 5 °C to 50 °C. Measurements were performed at pH 5 (red line), pH 7 (black line) and pH 8 (blue line). The protein concentration was 200 μM

We also systematically tested the temperature dependence of the putative AB_*h*RXRB phase separation by measuring the fluorescence anisotropy of Atto-488-labeled AB_*h*RXRB (Fig. 10). Fluorescence anisotropy is related to rotational flexibility and increases during the formation of supramolecular assemblies because of the restricted mobility within the assemblies [74]. We tested AB_*h*RXRB at three different pH values (Fig. 10). However, none of these conditions allowed for us to observe LLPS formation.

**Fig. 11:**
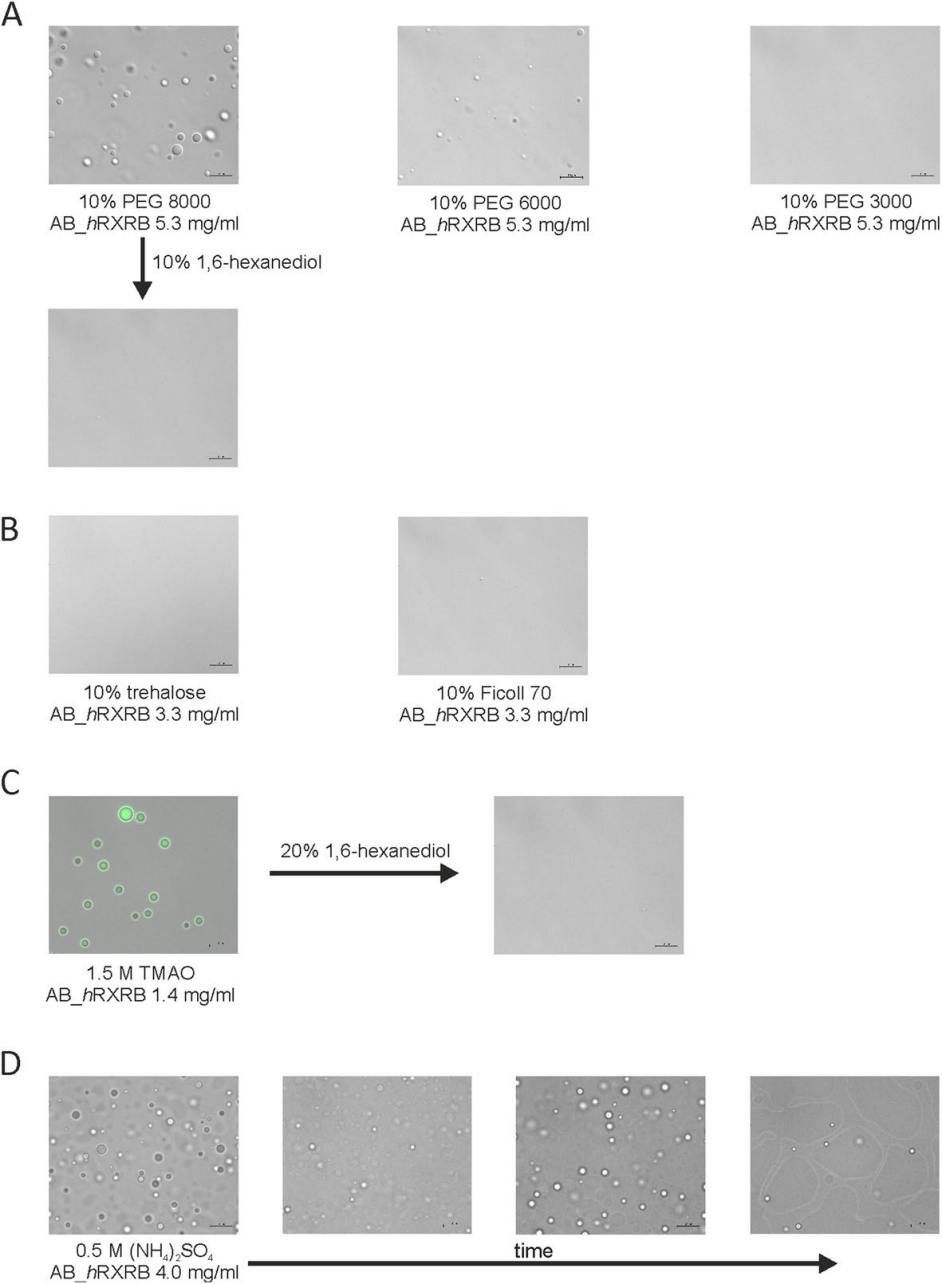
Factors influencing the formation of AB_*h*RXRB liquid condensates. **A** The phase behavior of AB_*h*RXRB in the presence of crowding agents. Representative images obtained by DIC of 260 μM AB_*h*RXRB in the presence of 10% (w/v) PEG 8000, 10% (w/v) PEG 6000 and 10% (w/v) PEG 3000. The addition of 10% (w/v) 1,6-hexanediol to the AB_*h*RXRB sample with 10% (w/v) PEG 8000 allowed for the droplets to dissolve. **B** The phase behavior of AB_*h*RXRB in the presence of 10% trehalose and 10% Ficoll 70. Representative images obtained by DIC of 165 μM AB_*h*RXRB. **C** AB_*h*RXRB droplet formation in the presence of TMAO. Incorporation of Atto 488-labeled AB_*h*RXRB (70 μM) into the droplets in the presence of 1.5 M TMAO. The addition of 20% (w/v) 1,6-hexanediol to the AB_*h*RXRB sample with 1.5 M TMAO dissolved the droplets. **D** The phase behavior of AB_*h*RXRB in the presence of kosmotropic salt. LLPS driven by 200 μM AB_*h*RXRB in the presence of 0.5 M (NH_4_)_2_SO_4_. The wetting properties of AB_*h*RXRB liquid droplets on the surface of a glass slide over time are also apparent. Scale bars: 10 μm

Finally, we decided to test the propensity of AB_*h*RXRB for the formation of liquid condensates in conditions that mimic the crowded intracellular environment. Analysis in a buffer supplemented with various macromolecular crowding agents was performed (Fig. 11A-C). First, we compared the propensity of AB_*h*RXRB for LLPS in solutions containing PEGs of different molecular masses (Fig. 11A). When AB_*h*RXRB was added to buffers containing 10% (w/v) PEG 8000 and 10% (w/v) PEG 6000, the formation of condensates by AB_*h*RXRB was observed and confirmed by turbidity measurements (data not shown) and DIC (Fig. 11A). Additionally, differences in the number and size of the droplets were observed (Fig. 11A). In the presence of 10% (w/v) PEG 8000, there were more AB_*h*RXRB droplets with sizes between 2–5 μm, and fewer in the presence of 10% (w/v) PEG 6000, and their size was reduced (1–2.5 μm). In the case of 10% (w/v) PEG 3000, we did not observe AB_*h*RXRB LLPS (Fig. 11A). These data show that the LLPS of AB_*h*RXRB is dependent on the molecular mass of the crowding agent. Moreover, the formation of condensates by AB_*h*RXRB in the presence of PEGs was observed after 5–10 min, depending on the concentrations of the protein and crowding agent (data not shown). The analysis of the AB_*h*RXRB sequence with the PSPredictor tool [51], which gives access to the most similar proteins (to the query protein) in the liquid–liquid phase separation database (LLPSDB) and their experimentally validated phase separation conditions, indicated that for most of the proteins (e.g., Mid1p, Shugoshin (Sgo), Intersectin-1 and son of sevenless homolog 1 (Sos1)), time was a crucial factor in the formation of condensates. The LLPS of AB_*h*RXRB was dependent on the molecular mass of PEGs as well as on the crowding agent used. We did not observe droplet formation in the presence of 10% (w/v) Ficoll 70 [75] (Fig. 11B). The formation of liquid AB_*h*RXRB condensates was also tested in the presence of 10% (w/v) trehalose (Fig. 11B). However, under these conditions, we were not able to detect droplet formation. We also verified AB_*h*RXRB LLPS in the presence of an osmolyte, trimethylamine N-oxide (TMAO) (Fig. 11C). After the addition of AB_*h*RXRB to buffer containing TMAO, the solution immediately turned opaque. LLPS of AB_*h*RXRB was confirmed by turbidity measurements (data not shown) and DIC (Fig. 11C). Additionally, using Atto 488-labeled protein, the presence of AB_*h*RXRB in the liquid droplets was confirmed. Altogether, the above results indicate that various crowding agents can have different impacts on the formation of AB_*h*RXRB condensates.

After confirmation of the propensity of AB_*h*RXRB for droplet formation, we decided to ascertain the type of interactions that are responsible for their formation. According to the Hofmeister series of salts [76], chaotropic salts such as GdmCl can weaken the stability of protein assemblies that are stabilized by hydrophobic interactions, whereas “neutral” salts, such as NaCl, or kosmotropic salts such as ammonium sulfate (NH_4_)_2_SO_4_ can promote hydrophobic interactions between proteins [77]. The formation of AB_*h*RXRB liquid condensates was prevented when chaotropic salts such as GdmHCl were applied (data not shown). On the other hand, when a kosmotropic salt such as (NH_4_)_2_SO_4_ was added, AB_*h*RXRB liquid condensates appeared (Fig. 11D), which was confirmed by turbidity measurements and DIC. Additionally, after a few minutes, droplets settled onto and wetted the surface of the glass coverslip, where they remained immobile (Fig. 11D). To obtain further insight into the type of interactions that are responsible for AB_*h*RXRB LLPS, we investigated the sensitivity to 1,6-hexanediol, a compound known to disrupt liquid-like condensates by interfering with hydrophobic interactions [78]. We tested the effects of 1,6-hexanediol on AB_*h*RXRB liquid condensates in the presence of PEG 8000 (Fig. 11A) and TMAO (Fig. 11C). The turbidity measurements (data not shown) and DIC show that AB_*h*RXRB liquid condensates were dissolved completely upon the addition of 10% or 20% 1,6-hexanediol to the solution containing PEG 8000 and TMAO, respectively. Lower concentrations of 1,6-hexanediol only partially disrupted the AB_*h*RXRB liquid condensates (data not shown). The sensitivity of the AB_*h*RXRB liquid condensates to the presence of 1,6-hexanediol and kosmotropic salt demonstrates that hydrophobic interactions contribute to the LLPS.

**Fig. 12:**
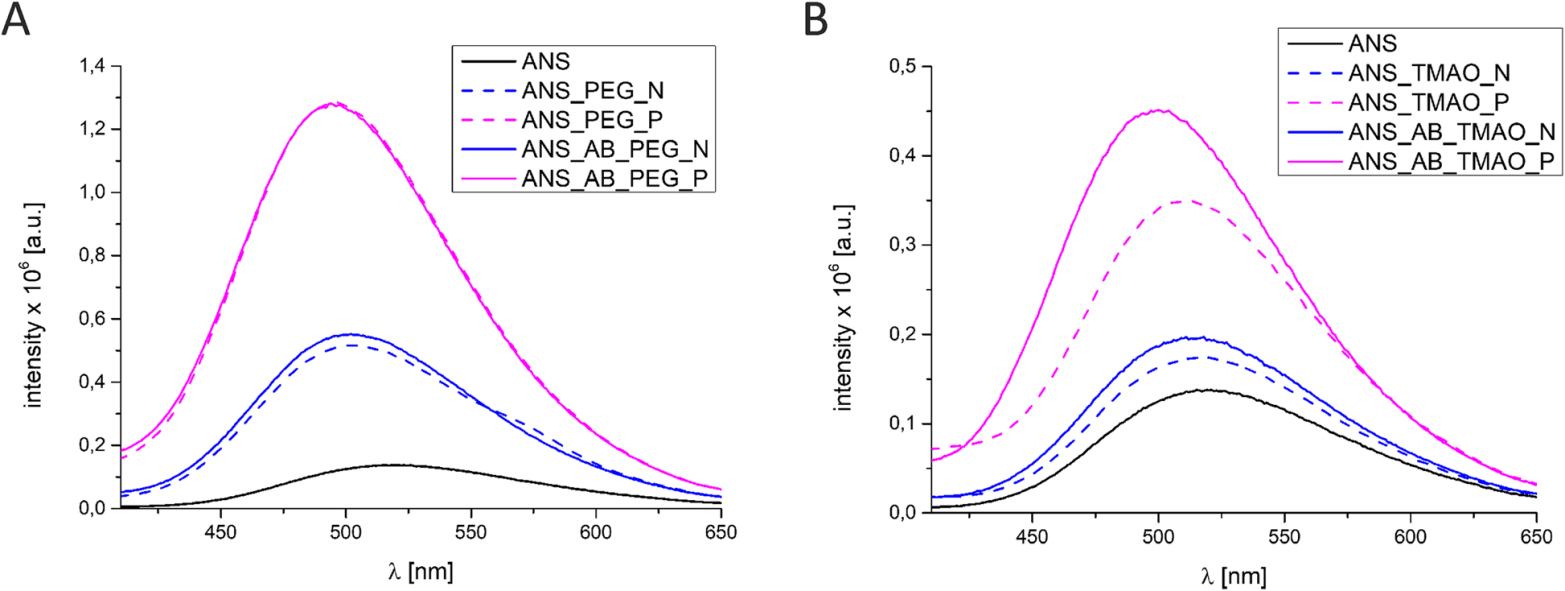
The effects of PEG 8000 and TMAO on AB_*h*RXRB. **A** Fluorescence spectra of ANS binding to AB_*h*RXRB in the presence of 5% (w/v) PEG 8000 (no LLPS (N), blue solid line) and 20% (w/v) PEG 8000 (LLPS (P), magenta solid line). The spectra of free ANS (black solid line) and ANS in the presence of 5% (blue dashed line) and 20% (magenta dashed line) PEG 8000 are also presented. **B** Fluorescence spectra of ANS binding to AB_*h*RXRB in the presence of 0.5 M TMAO (no LLPS (N), blue solid line) and 1.5 M TMAO (LLPS (P), magenta solid line). The spectra of free ANS (black solid line) and ANS in the presence of 0.5 M (blue dashed line) and 1.5 M (magenta dashed line) TMAO are also presented

We also verified whether the formation of liquid AB_*h*RXRB condensates in the presence of (NH_4_)_2_SO_4_, TMAO and PEG 8000 is connected with conformational changes in AB_*h*RXRB. The content of the secondary structures of AB_*h*RXRB in the presence of these factors was investigated using far-UV CD (Fig. S5). The obtained CD spectra of AB_*h*RXRB in the presence of (NH_4_)_2_SO_4_ and PEG 8000 were identical for AB_*h*RXRB (alone) and were typical for proteins containing disordered regions. These CD data suggest that the protein remains largely disordered within liquid condensates induced by (NH_4_)_2_SO_4_ and PEG 8000. However, the obtained CD spectrum for AB_*h*RXRB in the presence of TMAO (Fig. S5) is different from those in the presence of (NH_4_)_2_SO_4_ and PEG 8000. Changes in the secondary structure in the presence of TMAO and the potential impact of hydrophobic interactions in droplet formation of AB_*h*RXRB prompted us to look for further structural changes and exposure of hydrophobic amino acid residues. We utilized 8-anilino-1-naphthalenesulfonic acid (ANS) fluorescence in the presence of both PEG and TMAO (Fig. 12) and investigated two concentrations for each factor: one driving AB_*h*RXRB LLPS (P) and the other not driving AB_*h*RXRB LLPS (N). Free ANS excited at 351 nm had a fluorescence spectrum characterized by a low emission maximum located at 525 nm. However, the ANS quantum yield increases and the emission maximum shifts toward the blue as the solvent polarity decreases or upon binding to hydrophobic surfaces of proteins [79]. As shown in Fig. 12A, the fluorescence intensity of ANS increased in the presence of 5% and 20% PEG 8000 regardless of the addition of AB_*h*RXRB. Stronger fluorescent signals were observed for higher PEG 8000 concentrations. Additionally, the position of the fluorescence maxima blueshifted from 525 to 501 nm. However, the changes in the spectra were due to less solvent polarity.

Analogous experiments were conducted in the presence of TMAO. As shown in Fig. 12B, upon addition of AB_*h*RXRB in the presence of 0.5 M TMAO, the ANS fluorescence intensity increased 1.5-fold, and the position of the fluorescence maxima blueshifted from 525 to 511 nm. Under these conditions, AB_*h*RXRB does not have a propensity for LLPS (N), which was confirmed by measurements of turbidity (A_340_) and DIC (data not shown). Additionally, there were differences in the ANS fluorescence spectra depending on the absence or presence of AB_*h*RXRB. The major increase in the fluorescence intensity was observed upon the formation of LLPS (P) by AB_*h*RXRB in the presence of 1.5 M TMAO (Fig. 12B). The position of the fluorescence maxima blueshifted from 525 to 503 nm due to association of the ANS probe with AB_*h*RXRB. These results can be explained by the structural rearrangement and the altered hydrophobicity of the AB_*h*RXRB surface in the presence of 1.5 M TMAO. Altogether, the above results indicate that AB_*h*RXRB LLPS driven by TMAO, but not PEG 8000, induces conformational changes.

## Discussion

Nuclear receptors (NRs) are transcription factors (TFs) controlled by small lipophilic ligands [80]. Given the wide variety of genes and processes controlled by NRs, their dysregulation contributes to numerous diseases. As they represent promising therapeutic targets [81], it is crucial to understand the mechanisms underlying their actions and ways in which to selectively modulate their activities. The retinoid X receptor (RXR) is unique among NRs in that it can form dimers with other NRs [15]. In humans, there are three subtypes of RXR (α, β and γ) that differ mostly in their AB regions (Fig. S1). The N-terminal sequence of NRs often lacks a well-defined three-dimensional structure and possesses properties of IDRs [82, 83]. Each of these sequences has a characteristic disordered pattern that contributes significantly to the specificity of NR action. Upon interaction with coregulators or transcription factors, they may assume a locally stable structure [84]. Subtype β of RXR has the longest AB region, which has not yet been characterized at the molecular level. To describe the biochemical and biophysical properties of the AB region of RXRβ (AB_*h*RXRB), we have elaborated and optimized a protocol for efficient expression and purification. To overcome the problems with the expression of recombinant protein that has a high content of P amino acid residues (25.7%) and enhance its solubility, the SUMO fusion system [29] was chosen. This enabled us to obtain a high level of expression of AB_*h*RXRB. Additionally, during the purification procedure, the SUMO tag was removed by SUMO protease, resulting in the production of native protein (without additional amino acid residues). High susceptibility to degradation of AB_*h*RXRB noticed during the purification procedure, an increased electrophoretic mobility and in silico results suggested that AB_*h*RXRB might be an IDR, as was earlier observed for AB_*h*RXRG (Table S1) [27]. A comparison of the amino acid composition of both AB regions showed that although they are deficient in order-promoting amino acid residues and abundant in residues characterized as disorder-promoting, the percentage of particular amino acid residues are different (Figure S2). The differences in composition are especially seen for P, S, R, T, Y and N residues. Furthermore, the CD analysis in the far-UV region indicated that AB_*h*RXRB and AB_*h*RXRG have spectra that are characteristic of random coil conformations and confirmed the presence of numerous disordered regions. Despite significant differences in the composition of amino acid residues, the quantitative analysis revealed that 54.5% ± 1.7 of the AB_*h*RXRB sequence was unordered, which was only slightly lower than the value obtained for AB_*h*RXRG (61.8 ± 3.7) (Table S1). None of these values matched the results obtained with the disorder predictors, which indicated disorder of more than 80% in both AB region sequences. The difference between these values may be a consequence of the hidden (not predicted by in silico analysis) structure-forming propensity. Both AB regions (AB_*h*RXRB and AB_*h*RXRG) exhibit properties of IDPs/IDRs, but they also possess a substantial but different amount of ordered secondary structure motifs (Table S1). Such a residual structure can be involved in many molecular recognition events and binding to the physiological target of a given protein, which might be important for AB regions function, as they harbor an activation function (AF1) region. The analysis of the hydrodynamic properties of AB_*h*RXRB and AB_*h*RXRG indicated that, in solution, they exist as monomeric proteins with an extended conformation. However, based on CD and AUC experiments, they were classified into two different groups of IDPs – AB_*h*RXRB has properties of coil-like IDPs, whereas AB_*h*RXRG PMG-like IDPs. These different molecular properties of both sequences might have functional implications e.g., influence LLPS behavior of both proteins.

LLPS propensity depends not only on the presence of particular amino acid residues, but also on the number of residues and their position. The amino acid composition determines the structure, interactions and material properties of proteins, including their propensity to undergo LLPS. For example, LLPS may be driven by cation–π interactions between positively charged residues such as R and K and aromatic side chains such as Y and F, π stacking interactions between aromatic side chains, mainly Y and F [50]. Additionally, G residues increase fluidity, while Q and S residues increase hardening [85]. Although for some amino acid residues it is possible to define the role and the conditions in LLPS phenomenon, for others this can be a challenge. Analysis of the amino acid composition of the AB_*h*RXRB sequence showed a high content of P amino acid residues. The P amino acid residue has unique physicochemical properties and confers unusual structural properties [86]. It is also one of the most disorder-promoting amino acid residues and is associated with IDRs/IDPs [87]. P-rich motifs (PRMs) are common across the proteomes of many species and appear in various groups of proteins, including cytoskeletal proteins, nucleic acid-binding proteins, transport proteins, and splicing factors [88]. They are common recognition sites for protein–protein interaction modules such as the Src homology 3 (SH3) domain, the WW domain, and the Enabled/VASP homology 1 (EVH1) domain [89]. The AF regions of some TFs (e.g., AP-2 and CTF/NF1) [90, 91] are known to be enriched in P amino acid residues and have been classified on this basis [92]. It has been shown that these regions may serve as adaptor elements that bring together various components of the basal transcriptional machinery and coactivators [92]. Recent studies highlight the importance of PRMs as drivers of LLPS [93–95]. The PRMs of EBNA2 transcription factor has been indicated to play vital role in condensate formation [94]. However, to show the role of P residues, all residues (the content of P residues in the sequence is 27.3%) were mutated. The LLPS of EBNA2, as in the case of AB*h*RXRB, is observed in the presence of crowding agent (10% PEG 10 000) and the addition of 1,6-hexanediol significantly reduces droplet formation. On the other hand, higher concentration (300 mM) of NaCl suppresses EBNA2 droplet formation, what was not observed for AB*h*RXRB. Although, EBNA2 and AB*h*RXRB have high content of P residues (27.3% and 25.7%, respectively), which probably contributes to conditions of droplet formation, other amino acid residues seem also be important modulators of LLPS. The alignment of the RXRβ amino acid sequences of selected vertebrates (*Homo sapiens* (Human), *Canis lupus familiaris* (Dog), *Mus musculus* (Mouse), and *Danio rerio* (Zebrafish) indicates that there is sequence conservation within AB regions of these receptors (Figure S6). Most aligned sequences (except *Danio rerio*) have high content of P residues and contain a polyA stretch in the AB region. These N-terminal sequences of RXRβ may exhibit similar intrinsically disordered properties and propensities to LLPS under similar conditions. This indicates that the LLPS propensity is rather conserved among subtypes β of RXR. Moreover, as PRMs can rapidly switch between binding partners, form multivalent complexes [92] and drive LLPS, the high content of P amino acid residues in AB_*h*RXRB may also be critical for the regulation of gene expression.

The LLPS paradigm provides a new framework to understand the mechanism underlying the physiological function of NRs. NRs that are known to undergo LLPS leading to the formation of MLOs or liquid droplets in vitro include AR [24], ER [26], GR [25], PPARγ [23] and RXRγ [27, 28]. In different NRs, particular regions or domains with unique biochemical natures present different phase separation potentials. It was previously shown that an intrinsically disordered AB region of *h*RXRγ (AB_*h*RXRG) drives LLPS [27], and the process is ionic strength- and temperature-dependent. The formation of protein LLPS can be mediated by a myriad of different interactions. The sensitivity of AB_*h*RXRG LLPS to high ionic strength, kosmotropic salts and 1,6-hexanediol indicates the contribution of hydrophobic interactions in the formation of liquid droplets [28]. Additionally, molecular crowding agents and TMAO enabled phase transition at lower AB_*h*RXRG concentrations (Table S1). Here, our experimental data show that AB_*h*RXRB also has a propensity to induce LLPS. Although the formation of AB_*h*RXRB droplets is observed in the presence of molecular crowding agents and TMAO, and AB_*h*RXRB liquid condensates are sensitive to the average molecular mass of crowding agents, as well as sensitive to kosmotropic salts and 1,6-hexanediol, as in the case of AB_*h*RXRG, the phase propensity of AB_*h*RXRB exhibits some unique features. First, in silico analysis indicated that AB*h*RXRB may have greater propensity to undergo LLPS than AB*h*RXRG [28]. AB*h*RXRB was even classified as a droplet-driver protein. However, our results showed the opposite. AB*h*RXRB is not able to form liquid droplets in the absence of crowding agents in contrast to AB*h*RXRG. Moreover, after addition of crowding agent, the system needed higher concentration of AB*h*RXRB and longer time to undergo LLPS. It seems that these AB regions can play a different role in the context of full-length protein. AB_*h*RXRG seems to be critical scaffold element for liquid condensates integrity whereas AB_*h*RXRB seems to be a co-scaffolds region with ability to LLPS only in the presence of another component. Second, AB_*h*RXRB droplet formation does not depend on the ionic strength or temperature. In the N-terminus of AB_*h*RXRB, there is a fraction of negatively and positively charged residues. Although, it is possible that there may be electrostatic interactions between positively patches of one molecule of AB_*h*RXRB and negatively of other, these intermolecular interactions are not sufficient to induce LLPS of AB_*h*RXRB. Further LLPS analysis confirmed the role of hydrophobic interactions in LLPS of AB_*h*RXRB and suggested the role of hydrophobic patch located in the middle of the AB_*h*RXRB sequence (Fig. 9A).

AB_*h*RXRB also showed a propensity to form liquid condensates in the presence of an osmolyte, TMAO. In contrast to PEGs, TMAO leads to conformational changes in AB_*h*RXRB what may be relevant to AB_*h*RXRB function as AB_*h*RXRB has a potential to interact with other proteins. The main mechanism of TMAO action assumes exclusion of osmolytes from the protein surface [96]. It can also induce the folding of proteins due to the unfavorable interaction of osmolytes with the protein peptide backbone [63]. Molecular dynamic stimulation reported that TMAO may have opposing effects on hydrophobic and charge–charge interactions [97]. Additionally, TMAO may also act as a crowding agent [98] or weaken hydrogen bonds between water molecules and proteins [99]. The CD data indicated changes in AB_*h*RXRB secondary structure in the presence of TMAO. Additionally, the ANS fluorescence experiments showed that TMAO-induced LLPS triggers changes in the surface hydrophobicity of AB_*h*RXRB. The observed increase in ANS fluorescence intensity in TMAO-treated AB_*h*RXRB strongly suggests the exposure of hydrophobic groups and their involvement in LLPS of AB_*h*RXRB. These observations can be explained by the formation of a polyproline II (PP II) helix that presents an easily accessible hydrophobic surface [100]. Although hydrophobic interactions seem to be important in the formation of liquid droplets by both AB_*h*RXRB and AB_*h*RXRG, the distinct response to the environmental conditions driving LLPS can impact the propensity for droplet formation between a particular subtype of RXR and might be an important element of RXR action.

## Conclusions

Transcription is a complex process involving a large number of macromolecules, yet there is still much unknown information. A new aspect of gene expression is the ability of some transcription factors to induce LLPS and MLO formation. RXR is one of 48 human nuclear receptors and is an important ligand-activated transcription factor that has a unique ability to interact with other representatives of the family. Its three subtypes differ mostly in the N-termini (AB regions). Our experimental data show that AB_*h*RXRB is a highly disordered region that exhibits a tendency to form locally ordered structures. Additionally, AB_*h*RXRB possesses the ability to exhibit LLPS, a phenomenon previously described for AB_*h*RXRG. Although both AB regions seem to be similar in terms of their ability to induce phase separation, they clearly differ in the sensitivity to factors driving and regulating LLPS. The distinct LLPS response to environmental factors driven by the unique amino acid compositions of AB_*h*RXRB and AB_*h*RXRG can have important implications for the transcriptional activity of particular subtypes of RXRs. Further studies will be necessary to disclose how AB regions of RXR, especially in the context of full-length protein, regulate gene expression through phase separation.

## Supplementary Information

**Additional file 1: Fig. S1.** Alignment of the three human subtypes of RXR amino acid sequences. **Fig. S2.** Percentage of amino acid residues in AB_hRXRB and AB_hRXRG. **Fig. S3.** Sedimentation profile of AB_hRXRB. **Fig. S4.** Das-Pappu phase diagram [43]. **Fig. S5.** CD spectra of AB_hRXRB in the presence of different factors driving LLPS. **Fig. S6.** Alignment of the RXRβ amino acid sequences of selected vertebrates.

**Additional file 2: Table S1.** Comparison of results and properties of AB_hRXRB and AB_hRXRG.
