# Supplementary material for: Phase separation propensity of the intrinsically disordered AB region of human RXRβ
Source: Cell Commun Signal. 2023 May 4;21:92. doi: 10.1186/s12964-023-01113-4 (PMC10157963; doi:10.1186/s12964-023-01113-4)
Supplement: Supplementary file 2 — Additional file 1: Fig. S1. Alignment of the three human subtypes of RXR amino acid sequences. Fig. S2. Percentage of amino acid residues in AB_hRXRB and AB_hRXRG. Fig. S3. Sedimentation profile of AB_hRXRB. Fig. S4. Das-Pappu phase diagram [43]. Fig. S5. CD spectra of AB_hRXRB in the presence of different factors driving LLPS. Fig. S6. Alignment of the RXRβ amino acid sequences of selected vertebrates. [file 12964_2023_1113_MOESM1_ESM.docx]

*Supplementary data*

**Phase separation propensity of intrinsically disordered AB region of human RXRβ**

Katarzyna Sołtys^1^, Andrzej Ożyhar^1^

^1^Department of Biochemistry, Molecular Biology and Biotechnology, Faculty of Chemistry, Wroclaw University of Science and Technology, Wybrzeże Wyspiańskiego 27, 50-370 Wroclaw, Poland

To whom correspondence should be addressed. Email:

katarzyna.soltys@pwr.edu.pl

**
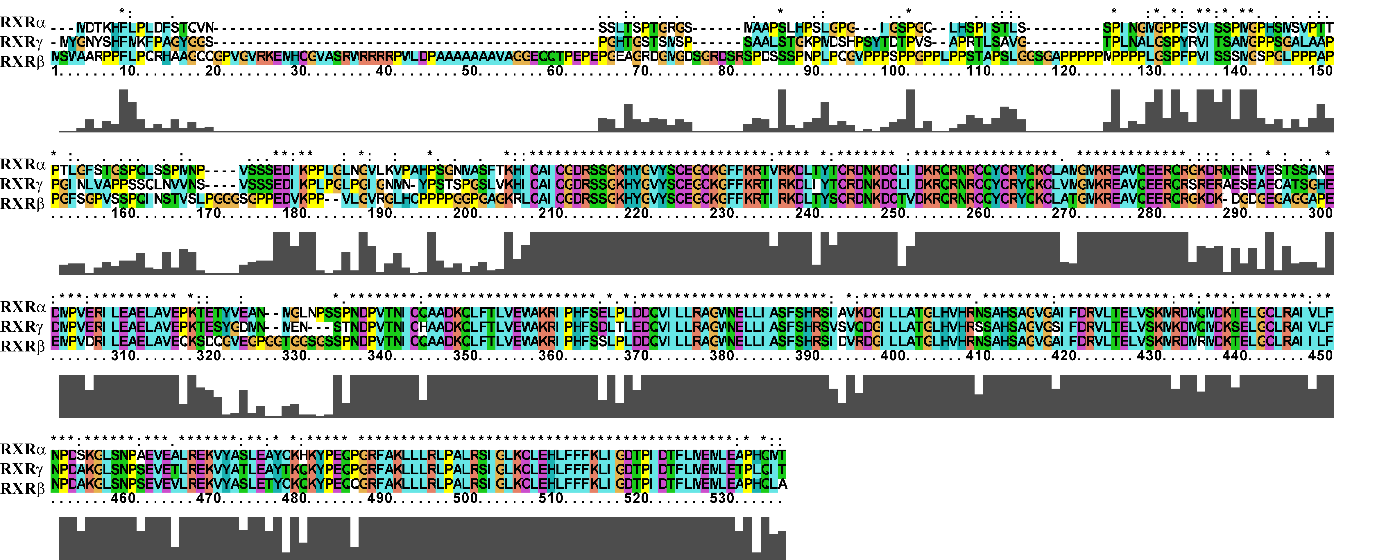
**

**Fig. S1. Alignment of the three human subtypes of RXR amino acid sequences.**

Amino acid alignment of sequences of human RXRα (UniProtKB accession number: P19793), RXRβ (UniProtKB accession number: P28702), and RXRγ (UniProtKB accession number: P48443). Sequences were aligned using the ClustalX 2.1 multiple alignment program [1]. In the line above each sequence, stars indicate amino acids that are conserved, while single and double dots denote amino acids that are similar in structure.

**
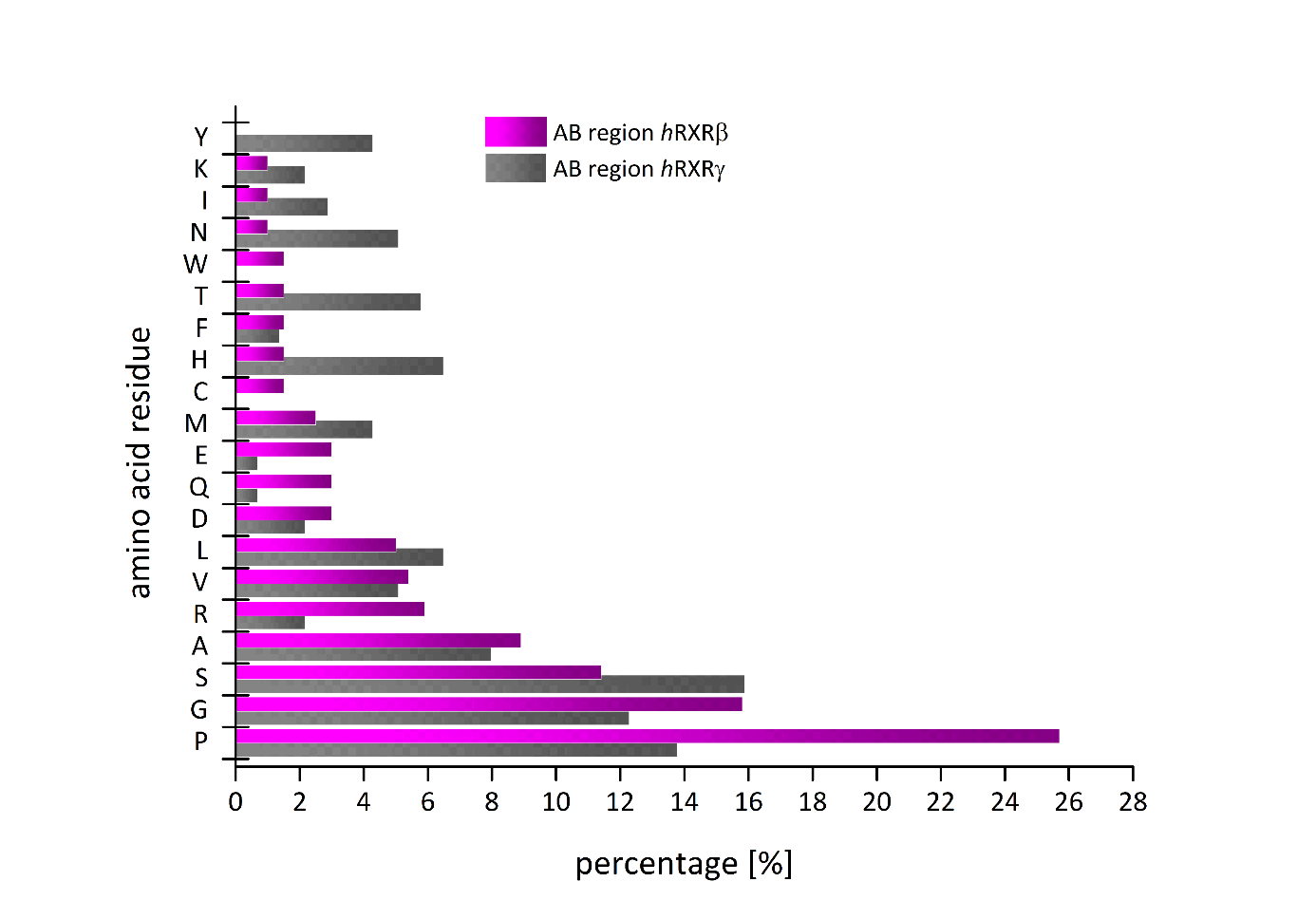
**

**Fig. S2. Percentage of amino acid residues in AB_*h*RXRB and AB_*h*RXRG.**

The comparison of amino acid residues composition of AB region of *h*RXRβ (1-202 amino acid residues) and AB region of *h*RXRγ [2]. The data were computed using ExPASy ProtParam tool [3].

**
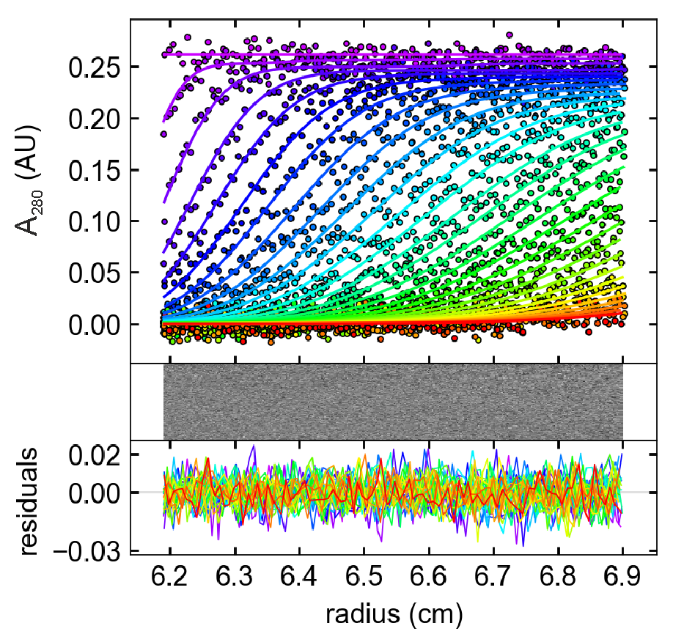
**

**Fig. S3. Sedimentation profile of AB_hRXRB.**

Superposition of selected experimental (circles) and fitted SV profiles (solid lines) corrected for systematic noises for AB_*h*RXRB at 0.25 mg/ml. The RMSD of 0.007598 indicates a good fit of the SV data. The lower panel shows the superposition of the differences between the experimental and fitted curves.


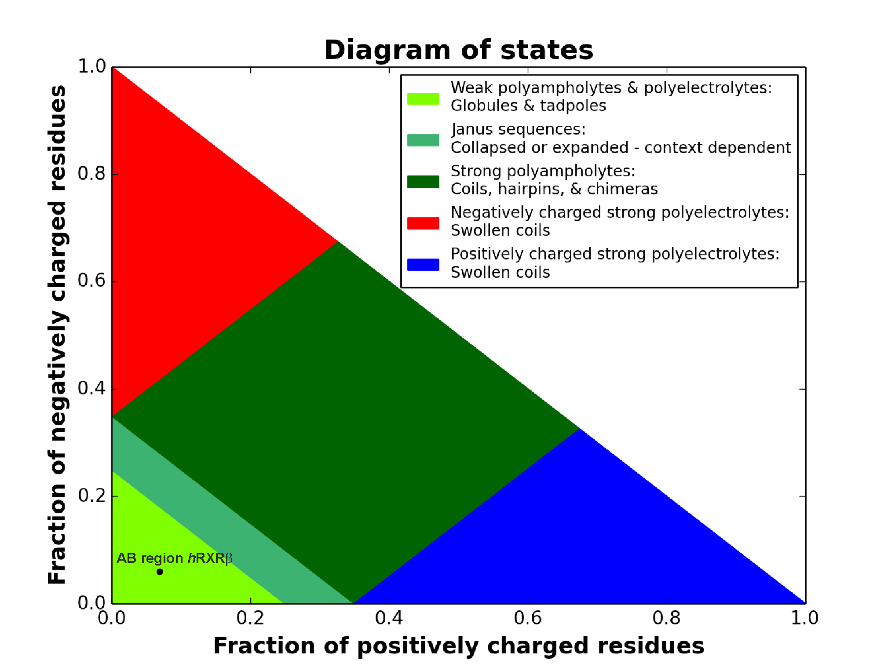


**Fig. S4. Das-Pappu phase diagram** [4]**.**

The diagram contains five regions (R1-R5) representing distinct conformational classes of IDRs. AB_*h*RXRB belongs to the group of weak polyampholytes.


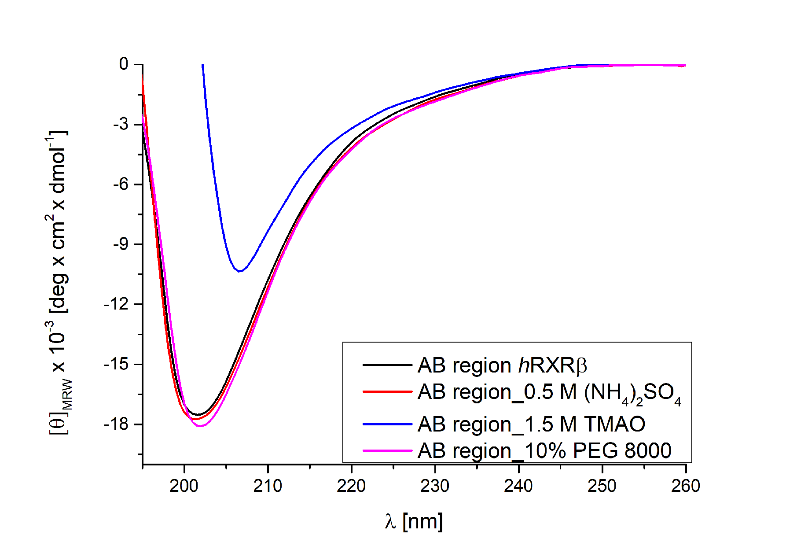


**Fig. S5. CD spectra of AB_*h*RXRB in the presence of different factors driving LLPS.**

The far-UV spectra of AB_*h*RXRB in the absence and presence of different agents (0.5 M (NH_4_)_2_SO_4_, 1.5 M TMAO and 10% (w/v) PEG 8000). The protein concentration was 10 μM (0.2 mg/ml), and the spectra were recorded at 20 °C.

**
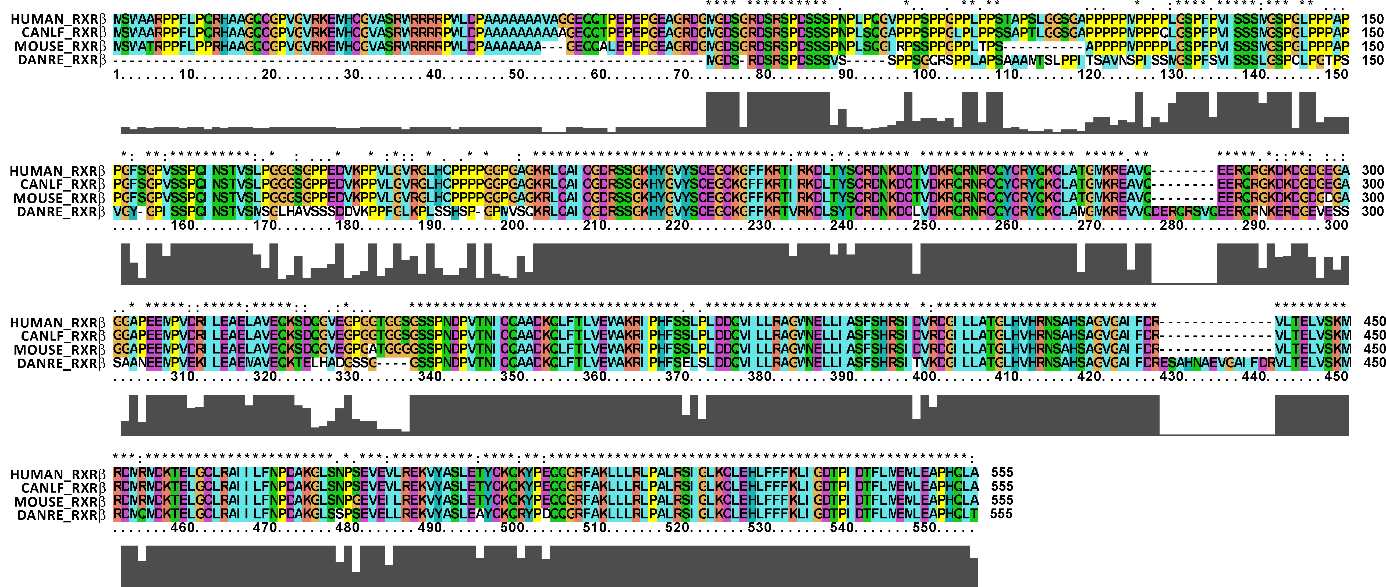
**

**Fig. S6. Alignment of the RXRβ amino acid sequences of selected vertebrates.**

Amino acid alignment of RXR subtypes β from *Homo sapiens* (HUMAN_RXRβ; UniProtKB accession number: P28702), *Canis lupus familiaris* (CANLF_RXRβ; UniProtKB accession number: Q5TJF7), *Mus musculus* (MOUSE_RXRβ; UniProtKB accession number: P28704), and *Danio rerio* (DANRE_RXRβ; UniProtKB accession number: Q7SYN5). Sequences were aligned using the ClustalX 2.1 multiple alignment program [1]. In the line above each sequence, stars indicate amino acids that are conserved, while single and double dots denote amino acids that are similar in structure.

**References**

1. Thompson JD, Gibson TJ, Plewniak F, Jeanmougin F, Higgins DG. The CLUSTAL_X windows interface: flexible strategies for multiple sequence alignment aided by quality analysis tools. Nucleic Acids Res. 1997;25:4876–82.

2. Sołtys K, Ożyhar A. Ordered structure-forming properties of the intrinsically disordered AB region of hRXRγ and its ability to promote liquid-liquid phase separation. J Steroid Biochem Mol Biol

3. Gasteiger E, Hoogland C, Gattiker A, Duvaud S, Wilkins MR, Appel RD, et al. Protein Identification and Analysis Tools on the ExPASy Server. In: Walker JM, editor. Proteomics Protoc Handb. Totowa, NJ: Humana Press; 2005. p. 571–607.

4. Holehouse AS, Das RK, Ahad JN, Richardson MOG, Pappu R V. CIDER: Resources to Analyze Sequence-Ensemble Relationships of Intrinsically Disordered Proteins. Biophys J. 2017;112:16–21.
