# Supplementary material for: Phase separation propensity of the intrinsically disordered AB region of human RXRβ
Source: Cell Commun Signal. 2023 May 4;21:92. doi: 10.1186/s12964-023-01113-4 (PMC10157963; doi:10.1186/s12964-023-01113-4)
Supplement: Supplementary file 3 — Additional file 2: Table S1. Comparison of results and properties of AB_hRXRB and AB_hRXRG. [file 12964_2023_1113_MOESM2_ESM.docx]

**Table S1. Comparison of results and properties of AB_hRXRB and AB_hRXRG.**

| **Type of analysis** | **AB_hRXRB** | **AB_hRXRG** [5] |
| --- | --- | --- |
| **amino acid composition** | dual character of the sequence (presence of both disorder- and order- promoting amino acid residues) | |
|  | high content of P (25.5%); no Y;  S (11.4%); R (5.9%); T (1.5%); N (1%) | P (13.8%); no C and W;  S (15.9%); R (2.2%); T (5.8%);  Y (4.3%); N (5.1%) |
| **in silico prediction of the degree of disorder** | Although 80% of analyzed sequence seems to be disordered, particular fragments of AB regions have various properties and scores of disorder, as well as different dynamic of protein backbone. | |
| **AUC** | monomer; coil-like IDP | monomer; PMG-like IDP |
| **CD** | total α-helix (%) 13.9 ± 2.0  total β-strand (%) 18.2 ± 2.0  turns (%) 16.7 ± 1.1  unordered (%) 54.5 ± 1.7 | total α-helix (%) 10.0 ± 1.0  total β-strand (%) 16.9 ± 2.0  turns (%) 12.9 ± 1.0  unordered (%) 61.8 ± 3.7 |
|  | coil-like IDP | PMG-like IDP |
|  | The AB_hRXRB undergoes a conformational change (rise of α-helix structure) in the presence of TFE.  Data for 30% TFE:  total α-helix (%) 20.4 ± 2.0  total β-strand (%) 12.7 ± 2.0  turns (%) 17.5 ± 1.3  unordered (%) 49.0 ± 3.5.  At 20-25% concentration of TFE, precipitation of protein is observed. | The AB_hRXRG undergoes a conformational change (rise of ordered structure with a prevalence of α-helix) in the presence of TFE.  Data for 30% TFE:  total α-helix (%) 20.5 ± 2.2  total β-strand (%) 18.1 ± 4.6  turns (%) 14.6 ± 0.8  unordered (%) 55.1 ± 3.9.  The change in the spectrum in the presence of 30% TFE is more visible compare to the AB_*h*RXRG alone than for AB_*h*RXRB.  At 15% concentration of TFE, precipitation of protein is observed. |
|  | Loss of the residual ordered structure in the presence of GdmCl. | |
|  | Both proteins exhibit properties characteristic of IDPs in response to increased temperature (increasing temperatures induce the formation of secondary structures). | |
| **limited proteolysis** | In the presence of osmolyte (TFE) the sequence is less sensitive to protease degradation. | |
| **LLPS propensity** | Higher total propensities scores for LLPS:  catGRANULE: 0.914  PScore: 4.30  FuzDrop: 0.9984  PSPredictor: 0.9913 | Lower total propensities scores for LLPS:  catGRANULE: 0.510  PScore: 2.91  FuzDrop: 0.9974  PSPredictor: 0.7584 |
|  | CIDER tool:  negatively and positively charged residues located mainly in the N-terminus of AB_*h*RXRB; hydrophobic patch in the middle of the AB_*h*RXRB sequence; weak polyampholyte | CIDER tool:  low proportion of charged residues; weak polyampholyte |
|  | LLPS of AB_hRXRB can be observed in the presence of particular crowding agents. | AB_hRXRG can lead to spontaneous LLPS via homotypic interactions in the absence of crowding agent. |
|  | AB_hRXRB does not undergo LLPS in response to changing NaCl concentration (75 – 500 mM). | LLPS at high NaCl concentration. |
|  | LLPS not temperature-dependent | LCST behavior |
|  | LLPS of AB_*h*RXRB is dependent on the used crowding agent – its concentration and molecular mass.  Higher concentration of AB_*h*RXRB is needed in comparison to AB_*h*RXRG.  Formation of liquid condensates is time dependent. | LLPS of AB_hRXRG is dependent on the concentration and molecular mass of crowding agent. |
|  | formation of liquid condensates in the presence of TMAO | |
|  | sensitivity to kosmotropic salt like (NH_4_)_2_SO_4_ | |
|  | sensitivity to 1,6-hexanediol | |
|  | The factors modulating LLPS of AB_hRXRB not allow to conduct experiment with deletion mutant of RXR that lack AB region. | AB_hRXRG can recruit deletion mutant protein that lack AB region into liquid condensates. |
|  | Hydrophobic interactions modulate LLPS of AB_hRXRB. | Mainly hydrophobic interactions drive LLPS of AB_hRXRG. |

**References**

1. Bolognesi B, Lorenzo Gotor N, Dhar R, Cirillo D, Baldrighi M, Tartaglia GG, et al. A Concentration-Dependent Liquid Phase Separation Can Cause Toxicity upon Increased Protein Expression. Cell Rep. 2016;16:222–31.

2. Vernon RM, Chong PA, Tsang B, Kim TH, Bah A, Farber P, et al. Pi-Pi contacts are an overlooked protein feature relevant to phase separation. Elife. 2018;7.

3. Hardenberg M, Horvath A, Ambrus V, Fuxreiter M, Vendruscolo M. Widespread occurrence of the droplet state of proteins in the human proteome. Proc Natl Acad Sci U S A. 2020;117:33254–62.

4. Chu X, Sun T, Li Q, Xu Y, Zhang Z, Lai L, et al. Prediction of liquid-liquid phase separating proteins using machine learning. BMC Bioinformatics. 2022;23:72.

5. Sołtys K, Ożyhar A. Ordered structure-forming properties of the intrinsically disordered AB region of hRXRγ and its ability to promote liquid-liquid phase separation. J Steroid Biochem Mol Biol. 2020;198:105571.
